# Assessment of Language Impairments Towards Identifying Markers for Early Diagnosis of Pathological Cognitive Decline

**DOI:** 10.3390/bs16030345

**Published:** 2026-02-28

**Authors:** Claudia Espinoza, Diana Martella

**Affiliations:** 1 Escuela de Psicología, Facultad de Ciencias Sociales y Comunicación, Universidad Santo Tomas, Valdivia 509000, Chile; 2 Departamento de Psicología, Facultad de Psicología y Educación, Universidad Loyola de Andalucía, 41007 Sevilla, Spain; dmartella@uloyola.es

**Keywords:** cognitive markers, language impairments, cognitive decline, dementia, assessment techniques

## Abstract

A major challenge in research on cognitive decline and dementia is the identification of at-risk populations in the preclinical phase. In this context, there is growing interest in language markers as early indicators of cognitive impairment. Objectives: This study aims to identify early linguistic markers that may facilitate the detection of individuals at risk of cognitive decline and dementia during the preclinical stage. Additionally, it seeks to evaluate the effectiveness of various assessment techniques and instruments for detecting such language impairments. Methods: A systematic review was conducted in accordance with the PRISMA guidelines, encompassing studies published between 2014 and 2025. A total of 109 articles were included in the qualitative synthesis. Results: The findings indicate that syntactic–structural features—particularly complexity, discourse coherence, and global organization—together with acoustic parameters such as pause duration, exhibit a higher accuracy and predictive value for the early diagnosis of cognitive decline and its progression to dementia. Furthermore, narrative-based tasks analyzed through automated methods demonstrate significant advantages for the assessment of language impairments. Conclusions: The analysis of language markers—particularly through the examination of syntactic complexity, acoustic features, and automated narrative assessments—represents a promising and effective approach for the early identification of cognitive impairment and the prediction of subsequent dementia onset.

## 1. Introduction

The accelerated aging of the global population necessitates the identification of risks associated with the development of chronic diseases and aging-related factors, with a particular emphasis on the prevention of age-related conditions, including dementias. In this context, the World Health Organization (WHO) recommends the implementation of proactive measures during middle age, between 45 and 59 years, to promote a healthy lifestyle and manage risk factors with long-term preventive effects, thereby reducing the likelihood of neurodegenerative diseases in old age (World Health Organization, 2015).

Dementia, in particular, imposes a substantial burden not only on affected individuals but also on their families, healthcare and social service systems (Macoir et al., 2017). The number of people diagnosed with dementia is rising rapidly worldwide, with estimates suggesting that by 2050, approximately 152 million individuals will be living with this syndrome (Calza et al., 2021). The management of this growing at-risk population poses a significant challenge; therefore, early diagnosis could enable targeted interventions aimed at slowing disease progression or mitigating its impact (Shi et al., 2023).

There is broad consensus that the neurodegenerative process leading to dementia begins long before the onset of clinical symptoms. This prolonged prodromal phase represents a critical window for the development of pharmacological treatments and therapeutic interventions aimed at implementing disease-modifying strategies in preclinical populations (Calza et al., 2021; Lowry et al., 2021). Consequently, one of the primary objectives of current dementia research is to refine early screening methods and differentiate individuals with mild cognitive impairment (MCI) who will progress to dementia from those who will not (Meilán et al., 2020).

In this regard, various studies have suggested that early alterations in normative speech patterns and language processing may be detectable in the prodromal stages of dementia, such as MCI, emerging even before formal psychometric criteria for this syndrome are met and demonstrating greater sensitivity than other cognitive impairments in the initial phases of the disease (Asgari et al., 2017; Beltrami et al., 2018; Jokel et al., 2019; Mueller et al., 2016; Szatloczki et al., 2015). The assessment of language markers could play a crucial role in identifying the likelihood of progression to dementia (Asgari et al., 2017; Beltrami et al., 2018; Jokel et al., 2019; Mueller et al., 2016; Szatloczki et al., 2015).

Previous research has extensively examined the cognitive abilities of individuals with MCI and Alzheimer’s disease (AD); however, studies addressing the language characteristics of these groups remain limited and are often focused on English-speaking populations and on cognitive decline specifically associated with AD (Kaltsa et al., 2024b).

Despite the importance of identifying language markers for the early diagnosis of cognitive decline, there is currently no consensus regarding the metrics required to determine the reliability of a marker. It has been suggested that the number of times a given feature significantly discriminates cases from controls, compared to the number of times it yields non-significant results, as well as the frequency with which the feature is employed in studies regardless of its outcome, are useful indicators of its reliability (Richard et al., 2024). In this regard, research has acknowledged the value of various language impairments as valid markers of cognitive decline, utilizing them for the classification of MCI and AD. Reported accuracy rates range from 0.71 to 0.80 for distinguishing MCI from healthy controls (HCs) and from 0.80 to 0.98 for differentiating AD from HCs (De Looze et al., 2022; Nasreddine et al., 2005).

Although there is extensive literature on language assessment and its contribution to the diagnosis of pathological cognitive decline, and despite evidence demonstrating that speech analysis is sensitive to the early detection of AD (Chou et al., 2024), it remains unclear whether these findings can be generalized to other forms of dementia. Furthermore, the results show considerable variability, reflecting the diversity of language assessment methods employed, the languages evaluated, and the number of participants included in the analyses (Kaltsa et al., 2024a), complicating the comparison of results and the derivation of consistent conclusions, thereby highlighting the need to standardize criteria and methodologies in language research to enable conclusive judgments that provide concrete evidence for more accurate diagnoses and more effective interventions (de la Hoz et al., 2021).

In this context, the primary objective of this systematic review was to gather evidence on language impairments, their accuracy and their predictive value for the diagnosis of cognitive decline and progression to various types of dementia, as well as to identify the main techniques and instruments used for language assessment and their effectiveness.

The authors developed this review based on three hypotheses: certain linguistic features exhibit higher levels of accuracy in distinguishing individuals with cognitive decline from those with healthy cognition; some linguistic features have greater predictive value for tracking the trajectory from HC to MCI and from MCI to various forms of dementia; and specific assessment tools demonstrate greater effectiveness in detecting language markers.

## 2. Method

This study was designed as a qualitative systematic review of the literature, conducted in accordance with the PRISMA guidelines. The research protocol was registered in the PROSPERO repository under the code CRD42023490321. Original articles published between 2014 and 2025 in Spanish and English were initially considered, with English prioritized as the predominant language of modern science. Given the global impact of dementia and the increasing scientific output in other languages, the search was subsequently expanded to include publications in Chinese, Japanese, Italian, German, and French. The studies included participants aged 45 years or older diagnosed with dementia, MCI, subjective cognitive decline (SCD), or HCs, and focused on the assessment of language and speech impairments, their accuracy, and their predictive value for diagnosing cognitive decline and progression to dementia. Studies of a pharmacological or genetic nature were excluded, as were those involving participants with other neurological or neuropsychiatric conditions, including multiple sclerosis, amyotrophic lateral sclerosis, schizophrenia, and mood disorders (both bipolar and unipolar).

## 3. Results

### 3.1. Study Characteristics

**Figure 1:**
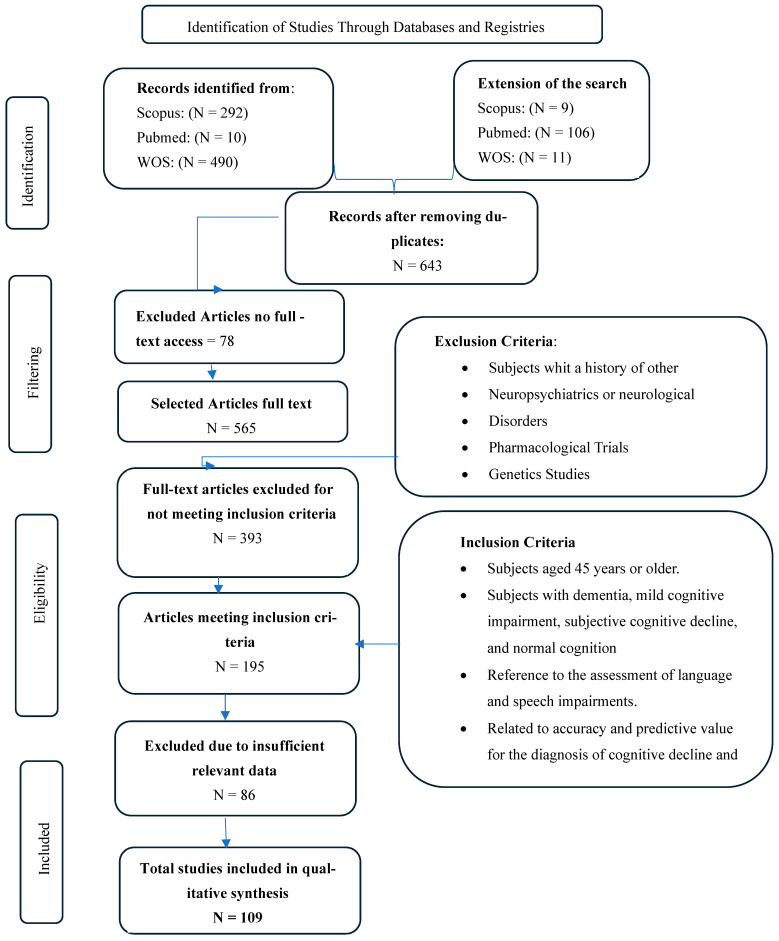
**Prisma flow diagram:** Flowchart of article retrieval process reported.

The literature search yielded a total of 918 articles, of which 109 were included in the qualitative synthesis. The process of identification, screening, and selection is detailed in the PRISMA flow diagram (Figure 1). A complete list of the articles identified through the search syntax and the consulted databases is presented (see Supplementary Table S1). The data extracted from each included article were summarized using a PICOS approach (see Supplementary Table S2).

The methodological quality of the studies was assessed using the “Standard Quality Assessment System,” a scoring system that provides a systematic, reproducible, and quantitative method for evaluating research quality and classifying it into four levels: low, moderate, good, and excellent (Kmet et al., 2004). The 109 articles included in the qualitative synthesis demonstrated good methodological quality, in accordance with the criteria of the Standard Quality Assessment System (see Supplementary Table S3).

Regarding sample characteristics, four of the included studies considered participants with SCD, defined as the perception of cognitive decline without objective deficits on standardized neuropsychological tests. Two of these studies also included an analysis covering the full spectrum from normal cognition to dementia, encompassing HC, SCD, MCI, early-onset Alzheimer’s disease (eAD), and AD.

The mean age of the participants was 67.5 years, with the majority being English speakers. Only 13 studies included native speakers of other languages, of which four considered Spanish, three French, two Swedish, and four included speakers of Chinese, Korean, Bengali and Persian. Of the 109 studies analyzed, 106 focused on cognitive decline associated with Alzheimer’s disease, two included participants with Lewy body disease, and one was on frontotemporal dementia.

### 3.2. Language Markers

Regarding language markers in cognitive decline—understood as observable features such as lexical, morphosyntactic, semantic, or pragmatic characteristics that can be used to detect patterns associated with neurocognitive disorders (Voleti et al., 2020)—40% of the studies (44 articles) emphasize the accuracy and predictive value of syntactic and structural features of speech, followed by acoustic parameters, which are reported in 19.09% of the studies (21 articles). The role of disfluencies, specifically filled and silent pauses, is highlighted in 15 studies, whereas 10 studies focus on lexical–semantic characteristics.

### 3.3. Language Assessment

Regarding language assessment tools—understood as a systematic process aimed at measuring an individual’s linguistic abilities through both formal and informal instruments—72 studies employed narrative tasks, of which 40 used picture description and 33 relied on personal or autobiographical narration. Twenty-three studies implemented verbal fluency (VF) tasks, including phonemic fluency (PF) and semantic fluency (SF), whereas twelve studies utilized reading tasks. Six studies incorporated naming tasks, and eight focused on conversational tasks. In addition, 27 articles applied automated techniques such as natural language processing (NLP), deep learning (DL), and automatic speech recognition (ASR) for the analysis of linguistic parameters.

## 4. Discussion

### 4.1. Language Markers for Cognitive Decline

The following section analyzes the findings derived from the qualitative analysis of the literature review in relation to language characteristics, precision, and predictive value as early markers for the diagnosis of cognitive decline and conversion to dementia.

### 4.2. Syntactic and Structural Characteristics

This category includes aspects related to syntactic complexity and the structural organization of speech, such as sentence length and complexity, the use of complex grammatical structures, coherence of speech, and the ability to maintain a logical sequence in narration. This set of characteristics was the most frequently mentioned in the literature, appearing in 40% of the studies included. While other research has noted that many grammatical and syntactical rules are preserved even in the later stages of AD (Kaltsa et al., 2024b). This review, consistent with findings from other authors, indicates that individuals with AD exhibit simplified syntax. They tend to produce shorter sentences with reduced structural complexity, affecting the overall accuracy of participants’ responses, and commit grammatical errors more frequently compared with cognitively healthy older adults. Recent studies have also suggested that this function declines even in the early stages of the disease (Can & Kuruoglu, 2019; Chou et al., 2024; Kaltsa et al., 2024a; Mueller et al., 2020).

In line with these findings, one study reported that sentence production was significantly impaired in MCI, suggesting weakened syntactic–semantic integration in the processing of complex sentences. The model employed demonstrated high accuracy in differentiating diagnostic groups and revealed that both core linguistic networks and auxiliary language networks are early targets of dementia-related pathology (Sherman et al., 2021). Syntactic complexity and cognitive decline have been shown to be significantly associated, with phonetic errors in the writing of ambiguous words, increased irrelevant information in written discourse, motor difficulties, and alterations in spatial organization highlighted, suggesting that ADD represents a clinically relevant measure (Sand Aronsson et al., 2021).

Furthermore, global coherence—defined as the ability to maintain a thematic thread and a logical, integrated organization in extended discourse or narration while minimizing irrelevant information—has also been highlighted as an important finding by other authors. Two studies comparing discourse measures in amnestic MCI (aMCI), non-amnestic MCI (naMCI), and cognitively healthy adults (HCs) found that the proportion of cohesive words (linguistic elements that establish cohesion, such as “this,” “that,” “another,” etc.) and propositional density (the number of ideas expressed per word unit in a statement) were lower in aMCI than in naMCI. Furthermore, subjects with naMCI performed worse than HCs in both cohesive word proportion and global coherence, even being outperformed by participants with aMCI in grammaticality and fluency (Kaltsa et al., 2024b; Kim et al., 2019).

Additionally, the study by Kaltsa et al. (2024a), which evaluated the linguistic abilities of elderly Greek individuals within the Alzheimer’s spectrum, ranging from normal cognition to SCD, MCI, and AD, using image-sequence narration, found that the macrostructure index (a measure that evaluates the global organization and essential content of a discourse or narration) was the most sensitive for identifying healthy cognition. In their analysis, the authors noted that individuals with AD systematically produced more irrelevant content in their narratives, while cognitively healthy younger and older adults generated less filler content than those with SCD or MCI. They concluded that the production of irrelevant information during discourse increases significantly as cognitive decline progresses toward a state of dementia (Kaltsa et al., 2024a).

### 4.3. Acoustic Characteristics

Acoustic variables, encompassing the physical attributes of speech signals, such as fundamental frequency, formant frequencies, amplitude patterns, and suprasegmental prosodic features of speech, including intonation, rhythm, intensity, and duration (Rodríguez-Fandiño & Montes, 2021), have also been considered as potential language markers for cognitive decline, appearing in 19.09% of the included articles. The altered presentation of these features in pathological groups has led several authors to highlight their potential to differentiate eAD, MCI, and HC, with classification accuracy rates approaching 80% using acoustic characteristics (Ivanova et al., 2023; Martinez-Nicolas et al., 2021; Shah et al., 2021). Notably, syllabic duration and variability have been identified as potentially the most significant rhythmic parameters (Martinez-Nicolas et al., 2021; Shah et al., 2021).

Furthermore, Beltrami et al. (2018) report that acoustic characteristics, such as the duration of silent segments, speech segments, and phonation time, exhibit significant differences between study groups, enabling differentiation among eAD, naMCI, aMCI, and HC.

Other studies have highlighted the strong predictive value of verbal vocalizations, intensity, fundamental frequency (the speed of vibration of the vocal cords during sound production, perceived as the voice’s basic tone), the asymmetry of central coefficients in MEL frequency (features extracted from the speech signal to capture its spectral content in a way that resembles human auditory perception), shimmer (instability or fluctuations in the intensity of the voice), and jitter (cycle-to-cycle variability in the fundamental frequency of the voice) (Badal et al., 2024; Fraser et al., 2015; Lee et al., 2024; Noto et al., 2024). In this regard, it was concluded that shimmer is the most influential characteristic in predicting cognitive decline, specifically suggesting that the fine motor control required to maintain stable vowel production is particularly sensitive to cognitive status. However, the variability in the importance of shimmer characteristics across different vowels raises important methodological issues, indicating that certain articulatory configurations may be more sensitive to cognitive decline than others, and therefore the results should be interpreted with caution (Lee et al., 2024).

Acoustic parameters have also been used in combination with other features to improve the discriminative power of classification models. A combination of acoustic and linguistic features (such as speech amount and vocabulary richness) significantly enhances the ability to distinguish between individuals with MCI and HC, with the potential to predict future cognitive performance in both initial assessments and follow-ups (Badal et al., 2024; Chou et al., 2024). Furthermore, the melodic contour of speech, defined as the appropriate use of intonation, volume, and duration, has also been analyzed, with significant increases in anomalies as cognitive decline progresses. It has been noted that lower speech volume is associated with the pre-dementia stage, and women exhibit greater loss of voice intensity (Mueller et al., 2018a; Meilán et al., 2020).

Despite these findings, some authors have pointed out that acoustic measures do not reflect optimal levels of accuracy, achieving only 67% to 76% in discriminating between HCs and individuals with MCI and AD (Huang et al., 2024), and 75% in studies aiming to predict amyloid status, without being able to clearly differentiate between groups (García-González, 2024; Xu et al., 2025).

### 4.4. Disfluencies and Pauses

It has been noted that the speech of individuals with MCI is characterized by longer production times due to stuttering, articulatory disfluencies, hesitations, and prolonged pauses, alongside a reduced speech rate (Meilán et al., 2020). Regarding pauses, the literature identifies two types: silent pauses, which reflect silent segments of speech production between statements, and filled pauses, which indicate hesitation by the speaker (Sluis et al., 2020). Several authors have pointed out that pauses in AD become longer, reducing speech rate and showing statistically significant impact in differentiating between the naMCI group and the HC group (Chou et al., 2024; Kaltsa et al., 2024a; Kim et al., 2019; Lofgren & Hinzen, 2022).

In 15 of the included studies, the value of pauses as a valid marker for identifying pathological groups was emphasized. The observed differences were not limited to their quantity but also encompassed their frequency and duration. For instance, in the study by Pistono et al. (2016), which included 15 individuals with MCI and 15 controls, participants with MCI did not produce more pauses overall but exhibited more frequent and prolonged pauses between phrases compared to controls. Similarly, in the study by Pastoriza-Domínguez et al. (2021), which characterized pause duration in patients with AD, MCI, HC, and MCI patients produced significantly longer and more variable pauses than the other groups, suggesting that the voice–silence ratio could serve as a reliable feature for differentiating narratives between MCI patients and HC. These findings have been corroborated by other authors, who reported that abnormal or prolonged pauses exceeding two seconds in narrative speech were significantly more prevalent in the AD group (Chou et al., 2024; Sluis et al., 2020; Wang et al., 2022).

Filled pauses, characterized by word repetitions, circumlocutions, and revisions, have been less frequently described in MCI and AD. However, it has been suggested that features such as empty speech, marked by an increase in word repetitions, occur more frequently in individuals with AD than in controls during image description tasks, indicating that these features may serve as markers of moderate to severe stages of AD (Mueller et al., 2020). Additionally, filled pauses in speech can help identify conversational difficulties in social interactions. A study on pause profiles in conversational speech of individuals with cognitive decline and HC found a progressive increase in these pauses in the more impaired groups (Ostrand & Gunstad, 2021).

### 4.5. Semantic and Lexical Features

These features are related to vocabulary, lexical richness, accuracy, semantic fluency, and content density in spontaneous speech, and were identified in ten of the studies included in this review as significant parameters in the early stages of dementia. A consistent reduction in content density was observed, associated with a global impoverishment of speech production from a lexical perspective, characterized by a decreased use of modifiers (e.g., adjectives), resulting in impoverished or empty speech. This has been described as a distinctive feature of AD (Beltrami et al., 2018; Kim et al., 2019; Momota et al., 2023). Some authors have even suggested that this may be the only domain capable of distinguishing between intermediate or slow-converting MCI cases and stable MCI cases that progress to dementia (Alegret et al., 2018; Mueller et al., 2020; Quaranta et al., 2023; Yamada et al., 2022). In this regard, a study including 696 participants assessed at five different time points found that lexico-semantic features were significant predictors of conversion. Another study, which included 20 individuals with SCD, 20 with MCI, and 20 HCs, reported impaired verbal production and difficulties in verb retrieval among the SCD group, suggesting that this condition may represent a precursor state to MCI, and that verb retrieval difficulties could serve as a marker of the preclinical stages of AD, given the higher cognitive demands and the more complex argumental and morphological structure of verbs (Alegret et al., 2018; Macoir et al., 2019; Mueller et al., 2020; Quaranta et al., 2023).

On the other hand, the contribution of the proportion of articles, pronouns, and nouns in differentiating AD patients from HCs has shown accuracy values ranging from 80% to 81.92% (Fraser et al., 2015; Mueller et al., 2018a; Orimaye et al., 2017; Ostrand & Gunstad, 2021). Of particular interest are the findings of Bayat et al. (2024), which suggest that these linguistic features do not arise from disruptions of language-specific morphosyntactic norms. In their study, the authors compared native English and Persian speakers, demonstrating that linguistic indicators of AD identified in English—such as reduced use of adjectives, increased use of pronouns and demonstratives, and decreased use of numerals—could be used to construct a classification model in Persian with an accuracy of 92.3%. The high degree of cross-linguistic transferability of these linguistic indicators from English to Persian strengthens the validity and reliability of these features as markers of cognitive decline (Bayat et al., 2024).

### 4.6. Evaluation of Language Markers

#### 4.6.1. Narrative Tasks

Currently, there is a wide variety of tasks available for analyzing narrative discourse, including interviews, picture descriptions, storytelling, as well as structured experimental tasks such as sentence completion, repetition, production of restricted sentences, and object naming (Diaz-Asper et al., 2022). One of the most used narrative tasks is picture description, considered in 39 studies as an effective method for obtaining a speech sample and evoking lexical retrieval. Among these, the most used stimulus for picture description is the “Cookie Theft” picture from the Boston Diagnostic Aphasia Examination (BDAE).

On the other hand, 33 of the included studies utilized expository narrative tasks, such as recounting a recent dream or describing a typical workday, emphasizing the benefits of autobiographical storytelling for its ability to represent a more challenging type of discourse than mere description, as it requires planning around a topic and reflects social and functional communication skills, providing a more ecological and real-life assessment compared to picture description tasks (Beltrami et al., 2018; Ostrand & Gunstad, 2021; Pistono et al., 2016).

#### 4.6.2. Verbal Fluency Tasks

These tasks appear in 23 of the studies and have been highlighted as a quick, relatively simple, accurate, and effective method for evaluating and monitoring cognitive skills, revealing significant discrepancies in performance between individuals at different cognitive stages (Lu et al., 2023). In a longitudinal study investigating the predictive value of an FV task for progression from normal cognition to MCI, the test showed a statistically significant decrease in performance as participants progressed from normal cognition to MCI and the early stages of AD. Healthy controls who progressed to MCI showed worse FV performance compared to those who remained stable, indicating a subtle gradient of decline, from normal aging to MCI and the early stages of AD (Alegret et al., 2018).

#### 4.6.3. Reading Tasks

Reading tasks were only present in 12 of the articles, where they were employed to analyze various parameters. For instance, in the study by Nagumo et al. (2020), which examined acoustic parameters through reading tasks in a large sample of controls and individuals with MCI, the reading tests differentiated control cases with moderate accuracy, achieving 77% precision. Additionally, Themistocleous et al. (2018) used a reading task to obtain speech samples and extract acoustic features, with the task providing meaningful information for identifying MCI and distinguishing it from controls, achieving high classification accuracy.

#### 4.6.4. Connected Speech Tasks

The analysis of connected speech, understood as spoken language produced in continuous sequences such as everyday conversations, could serve as an informative measure of language impairments from the mild to moderate stages of the AD continuum, being sensitive not only to early cognitive changes but also accurately reflecting real-life functional abilities (Mueller et al., 2018b). It has been suggested that the proportion of words spoken, disfluencies, and interactive features during a conversation are sensitive and ecologically valid indicators for identifying MCI and eAD, demonstrating a classification accuracy of up to 90% in some studies (Dodge et al., 2015; Nasreen et al., 2021; Mueller et al., 2018b; Noto et al., 2024). Natural language processing (NLP) techniques demonstrate promising diagnostic accuracy for the detection of cognitive decline across multiple languages and clinical contexts. However, the methodological heterogeneity and small sample sizes in existing studies indicate the need for larger, standardized investigations to establish their clinical utility (Shankar et al., 2025).

### 4.7. Automated Techniques

Finally, it is important to highlight that, in recent years, the use of automated methods has been proposed as an effective means to differentiate between healthy individuals and those diagnosed with dementia. These methods, particularly those based on DL and NLP, have shown potential for detecting subtle language changes (Calza et al., 2021). In a recent study that applied NLP to predict progression to AD over a 6-year period using speech features, the model achieved 78.5% accuracy and 81.1% sensitivity in predicting the progression from MCI to AD (Amini et al., 2024). Similarly, a voice interface using ASR, including tasks such as sentence pronunciation, reading, and word production, showed significant differences in the overall score between groups with cognitive impairment, with an AUC of 0.89, 90% sensitivity, and 82.9% specificity (Kong et al., 2024). Furthermore, a neural network model developed using artificial intelligence achieved an AUC of 0.84 in discriminating subjects with and without AD (Agbavor & Liang, 2023).

## 5. Conclusions

The authors conducted a qualitative systematic review with the aim of gathering evidence regarding language alterations as markers for the early diagnosis of cognitive decline and conversion to dementia, as well as the tools used for their assessment. The analysis was based on three hypotheses: 1. There are language characteristics with higher levels of precision in discriminating cognitively impaired individuals versus cognitively healthy individuals. Regarding this first hypothesis, the reviewed evidence indicates that there are differences between language characteristics, with syntactic–structural alterations, followed by acoustic features, being the most sensitive markers for the early detection of cognitive decline. The second hypothesis posited that there are language characteristics with higher predictive value for the conversion of cognitively healthy individuals to MCI and from MCI to dementia. In this regard, it was found that the coherence and global organization of speech, as well as the presence of irrelevant content in narratives, had greater predictive value for conversion to dementia, increasing significantly as the disease progresses. Regarding the third hypothesis, which proposed the existence of comparatively more sensitive assessment tools for detecting linguistic markers, it was observed that narrative tasks—including semi-structured and free narratives, such as those derived from autobiographical accounts or personal stories, as well as those arising from interactive conversational contexts—are particularly useful for accessing features that reflect a range of parameters, including lexical, grammatical, and acoustic aspects. These tasks also facilitate a more ecological evaluation of language.

Furthermore, it is important to highlight the role of automated procedures in language analysis, which are increasingly used in research, gradually replacing traditional methods of data recording and transcription. Methods such as DL, NLP and ASR are also notable for their ability to analyze language in natural and remote contexts.

### 5.1. Future Directions

In the future, research should focus on deepening the understanding and identification of language markers by differentiating linguistic profiles, as most of the studies analyzed concentrate on the Alzheimer’s disease spectrum, raising questions regarding the generalizability of the findings to other forms of dementia.

Furthermore, it would be highly valuable for future studies to incorporate factors such as monolingualism, bilingualism, sociocultural status, and literacy levels, to achieve greater precision in data analysis.

Finally, studies highlighting the ecological validity of certain tasks for language assessment present a significant challenge to current methods of cognitive evaluation. These findings suggest that future research should aim to incorporate measures with higher ecological validity, which better reflect patients’ real-life abilities, as these may be potentially more sensitive in detecting subtle and early changes in cognitive function.

### 5.2. Limitations

This systematic review is not without limitations, which are outlined as follows:

Publication Bias: Although the use of different search engines to identify relevant literature helped mitigate, to some extent, a biased or distorted view of the published findings, there remains the possibility that the review primarily accessed studies with positive results, excluding those with negative or null findings, thereby introducing bias into the conclusions of this review.

## Data Availability

No new data were created or analyzed in this study. Accordingly, data sharing is not applicable. All information supporting the findings of this study is contained within the article.

## Supplementary Materials

The following supporting information can be downloaded at: https://www.mdpi.com/article/10.3390/bs16030345/s1, Table S1: Identification of Articles by Search Engine and Syntax; Table S2: Key Findings from the Systematic Review; Table S3: Quality Scoring of Quantitative Studies.
