# Supplementary material for: Assessment of Language Impairments Towards Identifying Markers for Early Diagnosis of Pathological Cognitive Decline
Source: Behav Sci (Basel). 2026 Feb 28;16(3):345. doi: 10.3390/bs16030345 (PMC13024424; doi:10.3390/bs16030345)
Supplement: Supplementary file 1 [file behavsci-16-00345-s001.zip › Table S1.pdf]

Table S1: Identification of Articles by Search Engine and Syntax

Search Engine	Syntax	Articles Found
**WOS**	Language impairments, AND assessment	35
	Language impairments AND cognitive impairment	245
	Language impairments AND cognitive decline	87
	Language impairments AND older adults	78
	Language impairments AND middle-aged adults	4
	Language impairments AND early markers	39
	Language impairments AND predictive value	13
	Total, Search Engine	**501**
**Pubmed**	Language impairments, AND assessment	4
	Language impairments AND cognitive impairment	44
	Language impairments AND cognitive decline	56
	Language impairments AND older adults	6
	Language impairments AND middle-aged adults	6
	Language impairments AND early markers	0
	Language impairments AND predictive value	0
	Total, Search Engine	**116**
**Scopus**	Language impairments, AND assessment	98
	Language impairments AND cognitive impairment	108
	Language impairments AND cognitive decline	31
	Language impairments AND older adults	21
	Language impairments AND middle-aged adults	26
	Language impairments AND early markers	12
	Language impairments AND predictive value	5
	Total, Search Engine	**301**
