# Supplementary material for: Assessment of Language Impairments Towards Identifying Markers for Early Diagnosis of Pathological Cognitive Decline
Source: Behav Sci (Basel). 2026 Feb 28;16(3):345. doi: 10.3390/bs16030345 (PMC13024424; doi:10.3390/bs16030345)
Supplement: Supplementary file 1 [file behavsci-16-00345-s001.zip › Table S2.pdf]

Table S2: Key Findings from the Systematic Review

ID	Title	Author	Year of Publication	Sample	Tasks for Language Exploration	Results
**1**	Identification of Mild Cognitive Impairment from Speech in Swedish Using Deep Sequential Neural Networks	Themistocleous et al.,	2018	55 subjects: 30 healthy controls, 25 with MCI, mean age 69 years	Reading task, deep neural networks for acoustic feature analysis.	Acoustic features provide significant information for identifying MCI. Deep neural networks were able to classify controls and MCI with high precision. Individuals with MCI retain knowledge of morphological rules but face difficulties in processing pseudowords.
**2**	Discourse Measures to Differentiate Between Mild Cognitive Impairment and Healthy Aging.	Kim et al.,	2019	30 subjects with aMCI, mean age 73.80; 22 with naMCI, mean age 70.09; 21 healthy controls, mean age 71.90	Three discourse tasks: episodic narration, planning, and image description. Tasks were audio-recorded and orthographically transcribed.	Discourse measures indicate that both amnestic (aMCI) and non-amnestic (naMCI) MCI groups perform worse than controls, exhibiting lower global coherence, propositional density, and proportion of cohesive words, along with a higher presence of disfluencies and pauses. The proportion of pauses was the most discriminative measure between naMCI and controls, followed by the proportion of cohesive words. Both MCI subtypes show difficulties in conceptual organizational processing and greater reception of egocentric information during discourse production.
**3**	Deficits in narrative discourse elicited by visual stimuli are already present in patients with mild cognitive impairment	Drummond et al.,	2015	41 controls, mean age 69.6 years; 22 subjects with aMCI, mean age 72.1 years; 14 with AD, mean age 73.4 years.	Narrative task based on a story visually presented as a sequence of actions.	The control, aMCI, and AD groups differed mainly in discourse quality rather than quantity, with aMCI showing mild difficulties as an intermediate stage. AD and aMCI differed from controls in word type, within-sentence repetitions, narrative structure, and irrelevant propositions, while speech efficiency was the most discriminative parameter. Lexical measures and total words recalled showed no group differences. AD participants exhibited lower global coherence, more descriptive discourse, inadequate or ambiguous pronoun use, and impairments in semantic fluency (FS), whereas phonemic fluency (FF) remained unaffected.
**4**	Declines in Connected Language Are Associated with Very Early Mild Cognitive Impairment: Results from the Wisconsin Registry for Alzheimer's Prevention	Mueller et al.,	2018	264 participants at two time points: at visit 2, 64 with MCI and 200 with normal cognition. Mean age at visit 1: 61.8 years, mean age at visit 2: 64.2 years.	Image description: "The Cookie Theft" – the image was analyzed automatically.	There was a significant interaction between time and cognitive status, with aMCI participants declining faster than controls in semantic language (proportion of meaningful content words). Cognitive status did not predict syntactic performance or the decline in lexical diversity. However, it had a significant effect on fluency, with aMCI predicting poorer scores in disfluencies (repetitions, revisions, filled and unfilled pauses). Utterance length also differed significantly between aMCI and controls.

**5**	Analysis of word number and content in discourse of patients with mild to moderate Alzheimer's disease	Lira et al.,	2014	25 subjects with AD (divided into mild AD, ADg1, mean age 68.3 years, and moderate AD, ADg2, mean age 75.7 years) and 20 controls with a mean age of 71.1 years.	Description of the image The Cookie Theft.	The total number of words spoken by controls was significantly higher than in ADg1 and ADg2, with no differences between these two groups. The control group produced almost twice as many information units as ADg1 and more than twice as many as ADg2. There was a deficit in speech fluency in subjects with AD compared to healthy controls, but this impairment does not progress with the disease
**6**	An Automated Approach to Examining Pausing in the Speech of People with Dementia	Sluis et al.,	2020	60 English-speaking subjects between 53 and 88 years old: 20 with MCI, 20 with moderate cognitive impairment, and 20 healthy controls.	Image description	Progressive increase in pause duration across the three groups, respectively. Healthy adults follow predictable pause patterns. Significant differences in total pause duration between groups. Controls had fewer pauses than the MCI and moderate groups, and fewer pauses in MCI than in the moderate group. No significant differences in the number of pauses.
**7**	A Speech Recognition-based Solution for the Automatic Detection of Mild Cognitive Impairment from Spontaneous Speech	Toth et al.,	2018	28 healthy controls, mean age 64.13 years, and 48 with MCI, mean age 73.08 years.	Spontaneous speech was elicited through the recall of two black-and-white short films and by responding to a related question. Speech signals were recorded manually and automatically using an ASR system.	Significant differences were found in most acoustic parameters, with the most notable differences in speech rate and the number of pauses. The automated version using ASR in combination with machine learning differentiated the groups with an accuracy of 78.8%.
**8**	High amyloid burden is associated with fewer specific words during spontaneous speech in individuals with subjective cognitive decline	Verfaillie et al.,	2019	63 subjects with MCI, mean age 64 years, with positive amyloid status.	Image description: "The Cookie Theft," an abstract painting, and a random still life.	19 subjects with high BA load were moderately associated with a lower number of specific words, but not with syntactic and lexical complexity.
**9**	Neural correlates of spelling difficulties in Alzheimer's disease	Rodriguez-Ferreiro et al.,	2014	22 subjects with AD, mean age 75.3 years, and a control group with a mean age of 75.4 years, all	A battery that included image naming in writing, dictation of words and pseudowords, text dictation, and spontaneous writing.	The AD participants show a selective impairment in word dictation, in contrast to the writing of pseudowords. They had greater difficulty with words of arbitrary spelling and rule-based spelling. They also produced fewer complete syntactic units in spontaneous writing tasks compared to controls.

				native Spanish speakers.		
**10**	Pauses During Autobiographical Discourse Reflect Episodic Memory Processes in Early Alzheimer's Disease	Pistono et al.,	2016	15 with MCI, mean age 71.5 years, and 15 cognitively healthy controls, mean age 68.5 years.	Epitoul, an autobiographical memory test, was used to obtain samples of spontaneous speech.	MCI patients did not have more pauses than the controls, but they did make more pauses between sentences. The number of pauses correlated positively with the patients' episodic memory performance. The patients were less efficient than the controls in producing autobiographical discourse, producing fewer words per minute due to their longer pauses. They used more inter-verbal pauses than the controls.
**11**	Using Automatic Assessment of Speech Production to Predict Current and Future Cognitive Function in Older Adults	Ostrand et al.,	2021	39 subjects with mild or no language impairments.	Production of monologues based on image descriptions and expository tasks. Automated calculation of lexical-semantic characteristics. Follow-up one year later.	Lexical-semantic features were significantly associated with cognitive status both at baseline and one year later. Greater use of definite articles, determiners, and nouns correlated with better cognitive scores. Linguistic predictors from expository speech explained 56% of variance in future cognitive status, while features from image description explained 29%. The average lexical sequence was negatively correlated with future cognition, whereas the production of definite articles, nouns, and filler words showed a positive trend, particularly in the expository task.
**12**	Automatic Detection of Cognitive Impairments through Acoustic Analysis of Speech	Nagumo et al.,	2020	6,343 controls with a mean age of 73.5 years, 1,601 individuals with MCI with a mean age of 74.9 years. 367 participants with Global Cognitive Impairment, 468 with global MCI.	Four sentence reading tasks: vowel pronunciation, tongue twisters, diadochokinesis, and short sentences.	The model achieved moderate accuracy with an AUC of 0.77 for distinguishing global MCI from controls; the prediction score for differentiating MCI from global MCI remained low.
**13**	Connected Language in Late Middle-Aged Adults at Risk for Alzheimer's Disease	Mueller et al.,	2016	30 participants with aMCI and 39 controls. Mean age of 63.1 years, 22% women. The groups differed in terms of APOE ε4.	Connected language task using the description of the image The Cookie Theft. A 4-year follow-up from baseline, every 2 years.	Significant differences in content measures: controls produced more semantic units, more unique words, and higher idea density on average compared to aMCI. Verbal and learning scores were significantly different between the two groups, but not executive function. FF and FS showed lower scores in aMCI, while the BNT test was not significantly different between the two groups. The aMCI+APOE group produced fewer semantic units and unique words than the controls. No differences were found in syntactic complexity.
**14**	Proper names from story recall are associated with beta-amyloid in cognitively unimpaired adults at risk for Alzheimer's disease	Mueller et al.,	2020	696 subjects, mean age at second visit 58 years, mean age at the most recent With and without BA+.visit 65 years.	Story recall. 7-year follow-up.	Participants with BA+ were less likely to recall proper names in a story recall task during visit 1 compared to those without BA. Proper names, verbs, numerical expressions, and total score were significant predictors of conversion to clinical MCI.

**15**	Changes in the Rhythm of Speech Difference between People with Nondegenerative Mild Cognitive Impairment and with Preclinical Dementia	Meilán et al.,	2020	86 subjects, divided into non-demented MCI with high probability of non-degenerative MCI, and preclinical AD with a probability of developing AD..	Read-aloud task. Recorded.	The preclinical AD group showed longer speech duration and phonation time, more pauses, lower speech volume, monotonous intonation, and altered fluency compared to controls. Voice quality differed, with higher intensity in the global frequency spectrum, though vocal noise did not differ. Both groups exhibited clinical dysphonia. The main predictive features of preclinical AD were speech speed, phonation duration, and intonation.
**16**	Reduced Lexical Access to Verbs in Individuals with Subjective Cognitive Decline	Macoir et al.,	2019	20 subjects with subjective cognitive decline, mean age 66.4 years, 20 with MCI, mean age 71.05 years, and 20 controls, mean age 70.8 years	Naming tasks and fluency tasks. Naming of objects and actions, and free fluency. PF and SF.	The three groups performed worse in action naming than in object naming. In object naming, the SCD showed similar performance to controls, and both groups differed significantly from MCI. The total number of new words produced was significantly higher for objects than for actions in all three groups.
**17**	Discourse Measures to Differentiate Between Mild Cognitive Impairment and Healthy Aging	Kim et al.,	2019	30 participants with MCIa, mean age 73.80 years, 22 with MCIna, mean age 70.09 years, and 21 controls, mean age 71.90 years.	3 discourse tasks: episodic narration, planning, and image description.	MCIa participants performed worse than MCIna in the proportion of cohesive words and propositional density. Global coherence, the proportion of cohesive words, and the proportion of disfluencies and pauses in MCIna were lower than in controls. The proportion of cohesive words and pauses were common discourse measures for differentiating between MCIna and controls, as well as between MCIa and controls.
**18**	Word retrieval in picture descriptions produced by individuals with Alzheimer's disease	Kave et al.,	2016	20 AD subjects, mean age 76.60 years, and 20 healthy controls, mean age 76.20 years	Image naming tasks: The Cookie Theft and SF.	AD participants performed worse than healthy controls in the image naming and SF tasks, producing a lower proportion of content words overall, a lower syntactic proportion, and a higher proportion of pronouns, more frequent words, and pauses in the image description task. Total, word production was not significantly different.
**19**	Lexical-semantic ability in Alzheimer' disease: A study of verbal fluency with semantic categories	Ivanova et al.,	2020	126 controls, mean age 75 years; 48 MCI, mean age 79 years; 38 AD, mean age 79 years	FS task lasting >60 seconds, divided into four 15-second intervals.	Significant differences were found between the groups regarding lexical-semantic access capacity. The control group demonstrated a significantly higher lexical-semantic access capacity compared to individuals with MCI and AD, with both trends being statistically significant. In the control group and the MCI group, the decline in lexical-semantic access performance across the four intervals was statistically significant.
**20**	Naming and verbal learning in adults with Alzheimer's disease, mild	Hubner et al.,	2018	101 controls, age range 68–72 years; 17 MCI, age range 70–72 years; 19 AD, age	Naming of 60-line drawings (30 living and 30 non-living).	No significant differences in naming were found between the groups. The naming task distinguished between the healthy group and the two clinical groups with low education.

	cognitive impairment and in healthy aging, with low educational levels			range 72–73 years.		
**21**	Using narratives in differential diagnosis of neurodegenerative syndromes	Faroqi et al.,	2020	25 cognitively healthy English-speaking subjects, mean age 68.75 years; 20 with MCI, mean age 66.10 years; 20 with AD, mean age 68 years; and 26 with PPA	Image description task using The Cookie Theft picture, with automated speech samples	The PPA group scored lower than the healthy and MCI groups in fluency (words per minute and disfluencies) and sentence grammaticality. The AD and PPA groups did not differ in language measures. The sensitivity and specificity of the measures are modest and could be improved by combining them with clinical presentation.
**22**	Social Markers of Mild Cognitive Impairment: Proportion of Word Counts in Free Conversational Speech	Dodge et al.,	2015	83 participants divided into MCI and control groups, mean age 80.5 years.	Timed conversation	Compared to the controls, the MCI group generated a higher proportion of words during the timed conversation sessions.
**23**	Mild Cognitive Impairments Moderate the Effect of Time on Verbal Fluency Performance	Demetriou et al.,	2017	Subjects with a mean age of 76.41 years and a mean education level of 14.4 years, divided into 353 controls, 55 MCI, 13 aMCI, and 18 naMCI.	WRAT Reading Test, American National Adult Reading Test, PF, SF, BNT	The MCI group generated more words than the controls during the first 20 seconds of PF and SF. Group-time interactions revealed that individuals with MCI exhibited attenuated declines in word generation from the first second.
**24**	Language Changes in Late-Onset Alzheimer's Disease	Can et al.,	2019	39 patients with late-onset AD >65 years, and a control group.	Description of The Cookie Theft, description of the Picnic scene, image sequencing to tell a story, free speech test.	A greater number of sentences in AD compared to controls, but with less overall speech. AD patients produced more descriptive sentences and fewer sentences in free speech. Sentence length was longer in AD patients during the description of The Cookie Theft image and shorter in the Picnic description. Overall, AD patients' performance in terms of speech quantity was low.
**25**	Speech Analysis by Natural Language Processing Techniques: A Possible Tool for Very Early Detection of	Beltrami et al.	2018	48 controls with a mean age of 61.60 years, 48 with MCI, divided into 16 aMCI with a mean age of 64.19 years, 16 naMCI with a mean age of 64.50 years,	Description of a complex image, narration of a typical workday, narrating the last dream. Analysis with NLP.	Acoustic is the most affected linguistic category in the pathological group, including parameters of speech rate, pauses, and spectral properties of the voice. Significant impairment in early-onset dementia in lexical recognition and retrieval, decreased content density, and reduced use of adjectives.

	Cognitive Decline?			and 16 with early-onset dementia with a mean age of 66.38 years.		
**26**	The Role of Verb Fluency in the Detection of Early Cognitive Impairment in Alzheimer's Disease	Alegret et al.,	2018	568 cognitively healthy controls with a mean age of 63.3 years, 885 with MCI with a mean age of 71.7 years (535 aMCI and 350 naMCI), and 367 with AD with a mean age of 79 years.	VF of actions or verbs. BNT, PF, SF, Poppelreuter test.	A statistically significant worsening was observed from healthy controls to MCI and AD. Lower VF scores were significantly correlated with older age and lower educational level, but not with sex. Healthy controls who converted to MCI had worse baseline performance on the VF test compared to those who maintained stable cognition. The decline in VF with age was more pronounced in MCI and AD. VF showed a significant effect on faster conversion from healthy control to MCI, but not from MCI to AD.
**27**	Neural evidence for phonologically based language production deficits in older adults: An fMRI investigation of age-related differences in picture-word interference	Rizio et al.,	2017	20 young adults (18-31 years) and 20 older adults (60-79 years) all cognitively healthy.	Image-word interference paradigm. 240 color images, 60 unique items per condition, presented with an overlaid written word. The images featured common objects from various categories, including animals, clothing, food, and household items.	Main effect of age, as the naming accuracy task for younger adults was significantly better than for older adults. There was no significant interaction between the distractor condition and age. Younger adults had a significantly lower percentage than older adults.
**28**	Semantic intrusion errors as a function of age, amyloid, and volumetric loss: a confirmatory path analysis	Zheng et al.,	2022	212 participants ranging from normal cognition to dementia. 58% female, mean age 72.1 years.	Analysis of the relationship between proactive semantic interference and retrieval failure, in the presence of APOE ε4 genotype, amyloid positivity, and volumetric measurements. LASSI-L.	There is a direct relationship between amyloid positivity and decreased brain volumetric with intrusions in LASSI-L. The APOE ε4 status did not show direct effects on LASSI-L cognitive markers. Age did not have direct effects on LASSI-L scores.
**29**	Correlating natural language processing and automated speech analysis with clinician assessment to quantify speech-language changes in mild cognitive impairment and Alzheimer's dementia	Yeung et al.,	2021	Thirty audio recordings of subjects with MCI, AD, and controls.	Evaluation of difficulties in word finding, incoherences, perseverations, and speech errors. Linguistic and acoustic variables were extracted through NLP and ASA. "The cookie theft" picture description task was used. BDAE.	Difficulty in finding words was greater in AD and MCI compared to HC, with no difference between AD and MCI. Incoherence was greater in AD and MCI. Perseverations were greater in AD than in MCI, with no difference between MCI and HC. Speech errors were greater in AD than in HC, with no differences between AD and MCJ.

**30**	Comprehension of metaphors in patients with mild cognitive impairment: Evidence from behavioral and ERP data	Yang et al.,	2023	Thirty patients with MCI, with a mean age of 69.6 years, and 30 HC with a mean age of 65.3 years.	Evaluation of the ability to complete a final word sentence paradigm and judge the meaning of literal sentences, conventional metaphors, novel metaphors, and anomalous expressions.	Working memory (WM), semantic memory, and verbal fluency (VF) were positively associated with accuracy, which was highest for literal sentences, followed by anomalous expressions, conventional metaphors, and novel metaphors. Healthy controls outperformed MCI participants across all material types. Response times were longer for novel metaphors, while no differences were observed among the other sentence types. EEG N400 latency did not differ between groups. WM correlated with conventional metaphors and anomalous sentences; semantic memory correlated with conventional metaphors; VF correlated only with anomalous sentences. Overall, these cognitive variables explained 11.5% of the variance in accuracy.
**31**	A mobile application using automatic speech analysis for classifying Alzheimer? s disease and mild cognitive impairment	Yamada et al.,	2023	114 elderly participants, classified into 3 groups: AD (25), MCI (46), HC (43).	Collection of speech features, including acoustic, prosodic, and linguistic traits, through a mobile application and automatic transcriptions.	The machine learning speech classifier achieved an accuracy of 78.6% in classifying AD, MCI, and HC.
**32**	Speech and language characteristics differentiate Alzheimer's disease and dementia with Lewy bodies	Yamada et al.,	2022	121 subjects meeting diagnostic criteria for dementia. 45 with AD with a mean age of 73.1 years, 27 with LBD with a mean age of 75.1 years, and 49 controls with a mean age of 72.3 years. The AD and LBD groups included 25 and 19 subjects with MCI, respectively.	The subjects performed 5 speech tasks on a tablet: counting backward, subtraction, verbal fluency tasks (phonemic and semantic), and image description.	Participants with AD had reduced information units in the image description task and reduced responses in the VF task. In LBD, there were significant differences in phoneme speed, reduced tone variation, increased proportion of pause duration, and significant differences in acoustic characteristics.
**33**	Language dysfunction correlates with cognitive impairments in older adults without dementia mediated by amyloid pathology	Xiang et al.,	2023	272 participants aged 54-89 years, with MCI and healthy controls HC.	A follow-up of at least 3 years was conducted to assess the progression of language deterioration in semantic fluency and naming task.	SF and naming positively correlated with global cognitive decline and executive function decline specifically. Lower SF was associated with worse global cognition. Poorer performance in SF was significantly associated with a shorter estimated time to progression to MCI or AD. Greater decline in naming correlated with a higher risk of progression. Aβ pathology was significantly associated with lower levels of confrontation naming and SF.

**34**	Healthy ageing has divergent effects on verbal and non-verbal semantic cognition	Wu et al.,	2023	51 older adults with a mean age of 72.59 years and 48 young adults.	Completion of 3 types of semantic knowledge and control tests. Comparison of performance with young subjects. Test of Breadth of Semantic Knowledge (Picture), Test of Breadth of Semantic Knowledge (Word), Test of Semantic Control (Picture), Test of Semantic Control (Word).	The elderly were more accurate but slower than young subjects in image description and semantic control. Those with high performance in visual perception were more accurate in semantic tasks. Two different patterns of change throughout adulthood were observed for semantic knowledge and semantic control. The ability for controlled verbal retrieval remained in older adults but decreased in the non-verbal condition.
**35**	Screening for Mild Cognitive Impairment with Speech Interaction Based on Virtual Reality and Wearable Devices	Wu et al.,	2023	86 participants, 44 with MCI and 42 healthy controls aged 65 years or older.	Language task based on VR. Narrative task. Language task based on VR. Narrative task.	VR capable of capturing more ecological indicators of cognitive function. Greater use of complex sentences in HC. Subjects with MCI produced fewer characters in speech and spent more time exploring the VR environment. Both groups showed significantly different brain activity, with higher activity in MCI. The evaluation method achieved an accuracy of 89.8%.
**36**	Identification of Mild Cognitive Impairment among Chinese Based on Multiple Spoken Tasks	Wang et al.,	2021	50 patients diagnosed with MCI and 60 controls.	Completion of 3 spoken tasks: image description, semantic fluency, and sentence repetition. Training of machine learning classifiers.	SF tasks are more specific to controlled lexical retrieval processes. Repetition tasks reflect memory deficits.
**37**	Automatic Detection of Putative Mild Cognitive Impairment from Speech Acoustic Features in Mandarin-Speaking Elders	Wang et al.,	2023	41 older adults with MCI and 41 HC	Completion of 4 reading tasks: syllable enunciation, tongue twisters, diadochokinesis, and reading short sentences. Automatic extraction of acoustic features.	Temporal features (speech rate, duration of utterances, and number of silent pauses) and spectral and energy characteristics were effective predictors of MCI.
**38**	Speech silence character as a diagnostic biomarker of early cognitive decline and its functional mechanism: a multicenter cross-sectional cohort study	Wang et al.,	2022	324 participants, including 113 healthy controls with a mean age of 64.6 years, 95 individuals with MCI with a mean age of 73 years, and 116 in early-stage AD with a mean age of 76.4 years.	Analysis of PSD and the language-specific network involved in fragmented speech using task-based MRI.<br>BDAE.	PSD significantly increased in subjects with MCI and AD. This index was considered a valid biomarker to differentiate AD from HC. SF was associated with functional alteration of the language network. Combined language features achieved an accuracy of 80%. Greater activation of the Broca's area in patients with AD.

**39**	Semantic item-level metrics relate to future memory decline beyond existing cognitive tests in older adults without dementia	Vonk et al.,	2023	583 English speakers with a mean age of 76.3 years	Relationship of 7 SF metrics (one traditional, 4 psycholinguistic, two sequential) with semantic memory decline. Follow-up for up to 11 years. Repetition Test, BDAE.	Current standardized cognitive measures are not sensitive to early stages of AD. Robust and consistent relationship of 4 psycholinguistic metrics with memory decline. Total score of animal category SF was not related to memory decline.
**40**	Different language profiles on neuropsychological tests in dementia with Lewy bodies and Alzheimer's disease	Vogel et al.,	2023	90 patients with MCI, 77 patients with AD matched (MMSE score ≥ 21), and a group of 61 controls.	Through the BNT, famous face naming, and semantic and lexical fluency.	Patients obtained significantly lower scores on all tests compared to controls. Patients with AD obtained significantly lower scores than patients with MCI in naming measures, while lexical fluency score was significantly lower in patients with MCI. No significant differences were found in SF. The frequency of impairment in the BNT was higher in AD than in MCI, while the frequency of impairment in lexical fluency test was significantly higher in MCI.
**41**	Development of the Story Telling Examination for Early Mild Cognitive Impairment (Pre-Mild Cognitive Impairment) Screening	Taranop et al.,	2023	16 HC older adults aged 59-73 years.	Development of a Storytelling Task for STEEMS, an audio-based cognitive test.	13% of the subjects achieved 100% accuracy on STEEMS, 63% scored between 68% and 92% accuracy, and 25% scored between 40% and 60% accuracy. Correlation between STEEMS scores and MOCA scores.
**42**	The Joint Effects of Acoustic and Linguistic Markers for Early Identification of Mild Cognitive Impairment	Tang et al.,	2022	Audio samples from 160 older adults, half with normal cognition, average age 80 years, and the other half with MCI, average age 79.3 years.	WC and video chats VC. Semi-structured conversation with a predefined topic, extraction of acoustic and linguistic markers.	64 linguistic dimensions, 468 acoustic dimensions. 22 characteristics were positively correlated with MCI, and 42 obtained negative correlations with MCI. Linguistic categories with higher predictive value were leisure and money, correlating with a 30% decrease in the likelihood of MCI, while the categories death and home correlated with a 40% increase in the likelihood of MCI. Combination of acoustic and linguistic markers AUC 80%.
**43**	Automated semantic relevance as an indicator of cognitive decline: Out-of-sample validation on a large-scale longitudinal dataset	Stegmann et al.,	2022	1258 subjects without CD; average age 58.5 years, 180 with CD with atypical decline; average age 63.6 years, and 26 with MCI; average age 66.7 years, 195 with dementia 71.2 years.	Automatic and manual assessment of audio recordings on The Cookie Theft Picture Description Task from the Boston Test. BDAE	The three SemR versions were highly correlated with a low mean absolute error, showing that automatic SemR calculation reliably detects cognitive-linguistic changes. SemR correlated moderately with MMSE ($r = 0.38$), improving AUC from 0.78 (MMSE alone) to 0.81 (MMSE + SemR). SemR scores declined across all groups, with cognitively normal adults showing a slower decrease. Among impaired groups, decline was non-linear, highest in MCI, intermediate in mid-MCI, and lowest in dementia participants.

**44**	Inference comprehension from reading in individuals with mild cognitive impairment	Silagi et al.,	2021	100 individuals aged over 60 years, comprising 50 individuals with MCI, 35 with aMCI, 15 with naMCI, and 50 cognitively healthy controls	Inferential reading comprehension task via IMT, an implicit learning test evaluating 5 categories: explicit comprehension, logic, distractor, and pragmatics.	The MCI group showed poorer results in logical, pragmatic, distractor, and other questions. The aMCI and naMCI subgroups exhibited similar performance across all question types. Significant correlations were found between IMT total score and TMT-The aMCI group experienced a significant impact of memory on inference comprehension. The naMCI group had a greater impact of EFs on their performance. IMT proved useful in differentiating MCI from cognitively healthy individuals but was not useful in differentiating between aMCI and naMCI.
**45**	Alzheimer's Disease Diagnosis Based on a Semantic Rule-Based Modeling and Reasoning Approach	Shoaip et al.,	2021	2256 participants, 397 were diagnosed with AD, 561 diagnosed with late MCI, 389 were diagnosed with early MCI, 301 were diagnosed with SMC (significant memory concern), and 518 were in the NC group.	Identification of biomarkers through a semantic machine learning model.	The model achieved an accuracy of 92% for NC, 91.3% for SMC, 94.6% for early MCI, 93.4% in late MCI, and 92.6% in AD. Significant impact of the combination of biomarkers for classification. Age, education level, race, and marital status do not significantly influence the different classifications. Interestingly, age is a relevant factor in NC.
**46**	Language Decline Characterizes Amnestic Mild Cognitive Impairment Independent of Cognitive Decline	Sherma et al.,	2021	61 subjects with aMCI, average age 70 years, and 24 healthy controls, average age 67 years.	Psycholinguistic evaluation of complex sentence production. PA.	aMCI individuals showed significantly deficient sentence initiation compared to healthy adults and young individuals. aMCI individuals had difficulties in repeating challenging sentences involving syntax-semantics integration. The total ACE-R score significantly predicted performance in psycholinguistic tasks, yielding higher performance only in the healthy adult group.
**47**	Natural speech markers of Alzheimer's disease co-pathology in Lewy body dementias	Shellikeri et al.,	2022	22 with EA and LBD comorbidity and 38 without comorbidity, age range 66-78 years.	Automated analysis of natural speech, focusing on lexical-semantics and acoustic features of image descriptions. BNT, MDS UPDRS	Comorbidity results in an atypical language profile, with less detailed and more empty speech. Automated lexical measures are sensitive up to 95% in distinguishing comorbid cases from non-comorbid ones. Speech features perform well on their own without the need for cognitive tests; the latter did not improve discrimination.
**48**	Learning Language and Acoustic Models for Identifying Alzheimer's Dementia from Speech	Sha et al.,	2021	54 patients with AD and 54 healthy controls matched for sex and age.	Machine learning approach to distinguish between subjects with AD and controls, using acoustic and linguistic features of spontaneous speech via image description. BDAE	The best combination, acoustic + linguistic, outperformed the best independent language-based model. Language-based feature models achieved the best values, with 85% accuracy in classification.
**49**	Detecting impaired language processing in patients with mild	Sagaert et al.,	2022	23 patients with MCI, average age 70 years, and 23 age-matched	Detection of deficiencies, through electrodes, during a language comprehension task using a 2-word phrase paradigm.	Electrophysiological signatures were significantly different between MCI patients and controls. MCI subjects exhibit a deteriorated electrophysiological signature for understanding single words and multi-word phrases. Excellent sensitivity and

	cognitive impairment using around-the-ear cEEgrid electrodes			controls, average age 72 years. 29 healthy older adults, average age: 73.6 years; assessed with traditional EEG.		specificity of the single-word retrieval signature and electrophysiological signature to differentiate between cases and controls.
**50**	Screening for Mild Cognitive Impairment Using a Machine Learning Classifier and the Remote Speech Biomarker for Cognition: Evidence from Two Clinically Relevant Cohorts	Shafer et al.,	2023	121 participants, cases, and controls.	An algorithm KIKE SB-C was developed for detection based on speech biomarkers. PF.	The model that best differentiated between participants with MCI and healthy controls was a Support Vector Machine model. The algorithm's performance achieves similar results even when applied to a completely new and unknown dataset. The screening algorithm based on KIKE SB-C robustly detects MCI in a broad population and could be used to expedite recruitment for clinical trial enrichment.
**51**	Moving in Semantic Space in Prodromal and Very Early Alzheimer's Disease: An Item-Level Characterization of the Semantic Fluency Task	Sarampaa et al.,	2022	42 healthy volunteers, with a mean age of 74.4 years, 42 male participants; 24 with aMCI with a mean age of 71.34 years, and 18 with very early AD with a mean age of 78.32	Animal fluency task using the Word 2VEC method from the NLP domain. In the semantic fluency task, participants were asked to produce as many items from a specific semantic category as possible in 1 minute. The categories included animals, fruits, tools, and vehicles.	There was a significant difference between the groups in terms of the number of words produced. Healthy individuals named more animals than those with aMCI and AD. There were no differences between the groups in terms of the number of first-time produced words. Patients needed to return to semantic subcategories more times than controls.
**52**	The contribution of discursive and cognitive factors in referential choices made by elderly people during a narrative task	Sandoz et al.,	2023	78 participants aged 70-91 years, with a mean age of 75.3 years, French and Swiss speakers.	Narrative task (approx. 2 hrs.) in 3 stages of discourse: introduction, maintenance, change of referent or focus, at different levels of complexity (1 vs. 2 characters, different genders vs. same gender), recorded and transcribed verbatim. Zoo Map from the BADS, D-KEFS verbal fluency test, "Perspective Taking" subscale of the Interpersonal Reactivity Index	Both age and specific cognitive abilities play a role depending on the stage of discourse being referred to. Significant effect of discourse stages and complexity level. Higher likelihood of producing an indefinite marker in the introduction and change stages than in the maintenance stage. Significant interaction between referential complexity level and discourse stages as an effect of the presence of 1 vs. 2 characters. As age increases, the probability of a decrease in preferred pronouns in the maintenance stage also increases.
**53**	Is cognitive impairment associated with reduced syntactic complexity in	Sand Aronsson et al.,	2021	114 participants, 28 with subjective complaints, mean age 58.7 years; 41 with MCI, mean	Automated analysis using a syntactic parser and a part-of-speech tagger. BDAE	Significant association between ADD (syntactic complexity measure) and cognitive decline levels. Differences in idea density between early adulthood and late AD, with significant associations between low density and neuropathology.

	writing? Evidence from automated text analysis			age 62.5 years; and 45 with AD, mean age 67.4 years.		
**54**	Automated assessment of speech production and prediction of MCI in older adults	Sanborn et al.,	2022	88 subjects, 62 controls, 26 with MCI, average age 68 years.	Spontaneous speech task, to calculate lexical-semantic characteristics. Hopkins Verbal Learning Test, Controlled Oral Word Association Test, Animal Naming, Boston Naming Test – Short Form.	Higher predictive value for MCI with a combination of lexical-semantic features. Model sensitivity 7.7% (2/26 MCI cases classified correctly), specificity 95.2% (62 controls classified correctly), overall model accuracy 69.3%.
**55**	Speech Paralinguistic Approach for Detecting Dementia Using Gated Convolutional Neural Network	Makiuchi et al.,	2021	488 audio sessions, 169 subjects with dementia, 98 healthy.	Analysis of speech audios, extracting paralinguistic features.	Convolutional Neural Networks (CNN) method achieved an accuracy of 74.7% using 4 speech data segments and improved to 80% when using all data.
**56**	Using Digital Speech Assessments to Detect Early Signs of Cognitive Impairment	Robin et al.,	2021	75 participants, compared with 3 groups: 18 subjects scoring above the MOCA cutoff, with a mean age of 66.2 years; 18 subjects scoring below the cutoff with a mean age of 79.3 years, and 14 with MCI or AD, mean age 76.1 years.	The Winterlight Assessment (WLA) is a 6-task speech evaluation (picture description, reading, paragraph recall, semantic and letter fluency, object naming) recorded via microphone, with a total duration of 5–10 minutes, administered over a 6-month period.	Participants above the MoCA threshold performed better on speech measures of language coherence, information richness, syntactic complexity, and word-finding ability. The MCI and AD groups showed faster decline in language coherence over six months. Word-finding difficulties and accuracy of information units correlated significantly with baseline MoCA scores, whereas global coherence and syntactic complexity did not. Global coherence was the only measure showing a significant interaction, indicating a faster decline in the MCI/AD group compared to high-MoCA participants. Standard neuropsychological tests showed no significant change over six months. Word-finding and information accuracy had the strongest longitudinal associations, while global coherence and syntactic complexity showed moderate but significant correlations.
**57**	Automated detection of progressive speech changes in early Alzheimer's disease	Robin et al.,	2023	29 participants from the United States, English speakers, with a mean age of 68 years, meeting clinical criteria for probable AD, AD-related MCI, evidence of AD brain pathology confirmed by PET	Analysis of acoustic and linguistic measures including tone variability, speech rate, pause duration, word frequency, syntactic complexity using speech samples produced by patients during the Clinical Dementia Rating (CDR) assessment interview. Significant changes were identified over 18 months. ADCS-ADL	The speech composite score showed significant correlations with clinical scores and similar effect sizes for detecting longitudinal changes. The composite score demonstrated sensitivity to longitudinal changes in speech and linguistic patterns. Nine speech characteristics had significant temporal effects, suggesting constant and progressive changes during the study period. Six of these were linguistic characteristics representing word length and frequency, syntactic depth, noun usage, and pronoun-noun relationship.

**58**	Changes in the language system as amyloid-β accumulates	Reinartz et al.,	2021	35 cognitively healthy older adults aged between 65-71 years.	Analysis of functional changes in the language network in association with Aβ accumulation was conducted over a 10-year follow-up period, with tests administered every 2 years. AVLT, BART, Animal Fluency Test, Letter Fluency Test.	The main change was observed in the posterior temporal cortex, showing increased response amplitude during semantic associative tasks as amyloid deposition progressed over time. No significant correlation was found with reaction time in the confrontation naming task or lexical decision task.
**59**	The functional connectivity of language network across the life span: Disentangling the effects of typical aging from Alzheimer's disease	Rafiq et al.,	2022	23 healthy young adults, 24 healthy older adults with a mean age of 70 years, and 24 prodromal DA patients with a mean age of 73 years participated in the study.	The GREMOT evaluation assessed oral and written language, production, and comprehension across multiple levels, including phonological processing, naming, semantic and phonemic fluency, grammar, word and sentence repetition, reading, dictation, command execution, and syntactic comprehension. Narrative and discourse comprehension were also analyzed through an interview, with a total duration of approximately 2 hours.	Both groups of older adults exhibited lower connectivity values within frontal areas related to language. Healthy young and older adults scored higher than prodromal DA individuals in lexical-syntactic and phonological processing. Healthy older adults scored higher than young adults in famous person naming and word spelling. Both groups of older adults showed local functional decline.
**60**	Neuropsychological predictors of conversion from mild cognitive impairment to dementia at different timepoints	Quaranta et al.,	2023	253 subjects (129 females) with MCI, mean age of 72.95 years.	Tasks included phonological verbal fluency and CVF. ROCF, CDR, IADL, MFTC	186 progressed to dementia, while 67 remained stable. At the sixth year of follow-up, 27 progressed within the first two years (rapid converters), 107 in the third and fourth years (intermediate converters), and 51 after the fourth year of follow-up (slow converters). Those with stable MCI performed better than rapid converters in MMSE and categorial verbal fluency tests. Stable and slow converters differed only in MMSE and semantic phonological discrepancy score. Episodic memory measures significantly declined in rapid converters. Semantic lexical impairment was the only domain that significantly differentiated intermediate and slow converters from stable MCI after 6 years of follow-up.
**61**	Disfluency patterns in Alzheimer's disease and frontotemporal lobar degeneration	Pistono et al.,	2022	Participants with FTD had a mean age of 65.8 years, AD participants had a mean age of 67.9 years, and HC participants had a mean age of 67.7 years.	GREMOTs battery. Instructions were given: "This is a story represented in 5 pictures. Tell me the story with as much detail as possible."	Both the AD and FTD groups performed worse compared to the HC in fluency and naming tasks. Patients with FTD showed poorer performance in semantic and phonemic fluency compared to patients with AD. However, syntactic and phonological performance did not significantly differ between the groups after corrections for multiple comparisons.<br>Subjects with FTD, compared to AD, exhibited a lower speech rate and produced incomplete sentences. These measures did not correlate with linguistic abilities. Patients with AD did not differ from healthy controls in producing disfluencies. Discourse indicators correlated with the semantic lexical deterioration of the participants.

**62**	Language Network Connectivity Increases in Early Alzheimer's Disease	Pistono et al.,	2021	Right-handed native French speakers, 24 subjects with MCI due to AD, with a mean age of 72.9 years, and 24 healthy controls, with a mean age of 70 years.	They also underwent a computerized language assessment battery that evaluated both oral and written language, as well as production and comprehension at various levels of phonological, lexical, and syntactic processing.	MCI participants showed language impairments in standardized and connected speech tasks. Functional connectivity distinguished MCI better than executive control networks. AD participants produced more self-corrections and modulating discourse with lower lexical content, but similar repetitions and filled pauses as MCI. Gray matter-based classification achieved 95.8% accuracy ($p < 0.0001$), with key regions in bilateral frontal and temporal areas. Language network connectivity classification reached 64.5% accuracy ($p < 0.05$), showing increased connectivity in MCI due to AD. Higher lexical richness in controls correlated with differentiation from MCI-AD in connectivity ($r = 0.36$, $p = 0.015$).
**63**	Development of a machine learning model to predict mild cognitive impairment using natural language processing in the absence of screening	Penfold et al.,	2022	1473 participants from the ACT (Adult Changes in Thought) study and 2391 from the general population, attended at Kaiser Permanente Washington (KPWA) with an age range of 65-85 years.	Development of a natural language processing system based on clinical text. CASI	The performance of the prediction model on the validation dataset was modest with an AUC of 0.67%. The classifier yielded a sensitivity of 1.7% and a specificity of 99.7%, with a PPV of 70% and NPV of 70.5%.
**64**	Speech pause distribution as an early marker for Alzheimer's disease	Pastoriza-Dominguez et al.,	2022	112 participants: 26 subjects with AD, with a mean age of 81 years; 56 with MCI, and 29 HC with a mean age of 76 years. Patients in the AD group were significantly older (81 ± 6)	AD patients were assessed for pause density via manual transcription of 112 image-based oral narratives and duration extraction using a custom script. Additional tasks included a five-question personal/famous-person questionnaire, image description, BNT, category (animals) and letter (P) fluency, figure overlap test, and the Bilingual Aphasia Test.	Multi-domain MCI resulted in significantly longer and more variable pauses. The total number of pauses for each group was: 736 for HC, 679 for multi-domain MCI, 618 for MCI, and 669 for AD.
**65**	Language impairment in the moderate stage of dementia due to Alzheimer's disease.	Ortiz et al.,	2021	20 subjects with moderate-stage AD, with a mean age of 75 years, versus healthy controls.	Tasks of oral comprehension, speech tasks, reading comprehension, and writing. BDAE	Subjects showed impairment in all levels of linguistic processing, in oral and written comprehension and production. Regarding oral comprehension, word discrimination was equal to healthy controls. Compared to HC, AD exhibited poor performance in speech tasks. In visual confrontation naming and semantic cue tasks, no differences were observed between moderate AD and healthy subjects. In word and sentence repetition tasks, AD performed similarly to controls. Lower performance in AD versus controls in reading comprehension and writing. No differences were observed in sentence and paragraph reading.

**66**	Fully automated cognitive screening tool based on assessment of speech and language	O´Malley et al.,	2021	60 participants evenly divided into 4 groups: AD, with a mean age of 67.8 years; MCI, with a mean age of 63.4 years. Functional Memory Disorder (FMD), with a mean age of 54.9 years; HC, with a mean age of 69.5 years	Automated Cogno-Speak system development, where participants had to respond to questions posed by a virtual clinician.	The model achieved an accuracy of 87% in identifying AD and MCI, and 77% in correctly assigning HC and FMD. Overall correct classification was achieved in 65% of cases.
**67**	The copenhagen cross-linguistic naming test (C-CLNT): Development and validation in a multicultural memory clinic population	Nielsen et al.,	2023	186 participants, with 20 having affective disorders, an average age of 62 years; 67 with MCI, with an average age of 73 years; and 56 with dementia, with an average age of 74 years. There were also 43 control participants with an average age of 70 years.	BNT, and Positron Emission Tomography (PET). Participants were given one minute to produce as many different animal names as possible.	The test demonstrated acceptable reliability and good construct validity, with moderate to strong correlations with traditional language tests. Diagnostic accuracy for dementia was good, significantly better than the BOSTON test, with an accuracy of 80% and sensitivity of 75%. However, diagnostic accuracy for MCI was low.
**68**	Alzheimer's Dementia Recognition from Spontaneous Speech Using Disfluency and Interactional Features	Nasreen et al.,	2021	30 patients: 15 diagnosed with AD (4 men, 11 women) and 15 unaffected patients (4 men, 11 women). Patients with AD were aged between 60 and 89 years, while unaffected patients were of similar age.	Analyzing interaction characteristics during conversations using two sets of features: disfluency (self-corrections and fillers) and interactional features (overlap, turn-taking behavior, and distribution of silences).	The analysis achieved an 83% accuracy in classifying Alzheimer's and non-Alzheimer's conversations using disfluency features, and also 83% accuracy using interactional features. When combining both sets of features, the overall accuracy reached 90%

**69**	Amyloid beta associations with connected speech in cognitively unimpaired adults	Mueller et al.,	2023	255 middle-aged adult participants at risk of late-onset AD, of whom 73% had a probable family history of AD. Among them, 57 were Aβ+ and 198 were Aβ-, with an average age of 62.7 years	Speech samples were collected during biennial neuropsychological test visits, where participants described the "cookie theft" picture from the Boston Diagnostic Aphasia Examination. BNT PF from the Multilingual Aphasia	Aβ+ individuals experienced a faster decline in specific word meaning. However, there were no differences between the groups in lexical diversity measures over time.
**70**	A pilot screening for cognitive impairment through voice technology (WAY2AGE)	Moret Tatay et al.,	2023	30 volunteers aged over 60 years, classified into MCI with an average age of 75 years and SCD with an average age of 70 years.	The lexical access task involved responding to a voice-bot across seven items assessing orientation, lexical access, memory, attention, and calculation.	The WAY2AGE model showed a good Area Under the Curve (AUC) value for discriminating between groups with and without MCI. There was an inverse relationship between age and WAY2AGE scores, with a sensitivity of 79% and specificity of 90%.
**71**	Language patterns in Japanese patients with Alzheimer disease: A machine learning approach	Momota et al.,	2023	276 speech samples from 42 individuals with AD and 52 HC	Phonological, lexical, and morphosyntactic analyses were conducted to explore language patterns related to AD. Participants were tasked with describing an image. WMS-R	The model achieved an accuracy of 84%, with 7 discourse features and 3 dependency features contributing to prediction. The presence of language patterns characterized as empty speech, a type of semantic-lexical pattern associated with AD, was noted.
**72**	Lexical relations in Spanish-Speaking older adults with Alzheimer's disease: An approach to semantic memory	Minto-Garcia et al.,	2022	24 older adults, 12 with AD and 12 with TC (Typical Aging). Monolingual Spanish speakers, aged 55 or older.	Free word association task. 234 high-frequency nouns were used. Two codings were performed: 1. Based on syntagmatic and paradigmatic relationships. 2. Based on broad semantic association and association by signifiers	Differences were observed in the type of lexical relationships between AD and TC. Semantic memory in AD shows alterations in word connections, but diverse lexical relationships are preserved in the moderate stage of the disease.
**73**	Perceptual strength influences lexical decision in Alzheimer's disease	Miceli et al.,	2023	36 healthy subjects, 22 mild AD stage 1, 20 moderate AD stage 2.	The degree to which a word can be experienced through multiple sensory modalities was explored in visual word recognition. Lexical decision task with two conditions: words with high perceptual strength (PS) versus low PS. BREF, lexical-semantic tasks,	An interaction effect was observed only between healthy controls and AD stage 1, with the latter being faster in processing high PS words. Words with more sensorimotor knowledge generate higher semantic activation and favor faster responses.

**74**	Cognitive Writing Process Characteristics in Alzheimer’s Disease	Meulemans et al.,	2022	15 patients with MCI with a mean age of 74 years and 15 HC with a mean age of 74 years.	Analysis of pauses in writing, through tasks of describing typed images. Write a brief descriptive text for each of the two image description tasks: the cookie theft image from the BDAE and the situational drawing from the Dutch version of the Comprehensive Aphasia Test BDAE, Comprehensive Aphasia Test.	Pause time as a proportion of task time was 20.58% higher in MCI patients; additionally, they wrote fewer characters per minute, in more bursts, and with shorter episodes of interrupted writing.
**75**	Textual Inference Comprehension in Mild Cognitive Impairment: The Influence of Semantic Processing and Verbal Episodic Memory	Maziero et al.,	2021	99 subjects divided into 3 groups: 23 with aMCI (amnestic Mild Cognitive Impairment), mean age 70.6 years, mean education 11.6 years; 42 with naMCI (non-amnestic Mild Cognitive Impairment), mean age 69.7 years, mean education 11.6 years; and 34 healthy controls, mean age 70.6 years, mean education 14.4 years.	Analysis of textual inferences through the Implicit Management Test, reduced version, to assess various types of inferential. reasoning in text reading. FAS-COWA.	Subjects with aMCI performed worse than healthy elderly individuals, with no differences between the subgroups of aMCI. The best predictors for making inferences were verbal memory in the aMCI group and semantic tasks for naMCI. Education and tasks related to verbal episodic memory were the best predictors of performance in making inferences across different diagnostic groups.
**76**	Reading and lexical-semantic retrieval tasks outperforms single task speech analysis in the screening of mild cognitive impairment and Alzheimer’s disease	Martinez Nicolas et al.,	2023	72 native Spanish-speaking participants, divided into 3 groups: 24 healthy older adults, with a mean age of 82.5 years; 24 individuals with MCI with a mean age of 84.1 years; and 24 subjects	Speech production tasks, voice recordings. Reading of text and completion of sentences with semantic information. Screening, and verbal fluency, Free and Cued Reminding Test, BNT	Discriminative functions achieved an accuracy of 83.3%. Acoustic parameters (speech quality, rhythm, and duration) allow differentiation of the 3 groups. 2 significant rhythm parameters, related to duration and syllabic variability. Type of elicitation tasks and associated cognitive load are important factors for improving speech-based dementia detection.

				with AD, mean age 81.2 years		
**77**	Temporal Integration of Text Transcripts and Acoustic Features for Alzheimer's Diagnosis Based on Spontaneous Speech	Martin et al.,	2021	Voice samples from 156 participants, in a 1:1 ratio of AD and HC matched by age and sex.	Spontaneous speech analysis, exclusively using the cookie theft description task, where participants were asked to describe the image of "the cookie theft" from the BDAE	Using only semantic features achieved an accuracy of 89.58% in group distinction. When combining structural and temporal features, the accuracy was 91.67%. The test set achieved an accuracy of 97.75%. Semantic features and pause information contribute most to the method's performance and improve when including temporal and structural aspects of audio and text.
**78**	Speech Understanding in Modulated Noise and Speech Maskers as a Function of Cognitive Status in Older Adults	Mamo et al.	2021	39 participants aged 55-77 years old, 8 with MCI or Mild Dementia, mean age 70.8 years. 31 without a history of cognitive decline, mean age 66.4 years.	Speech task in the presence of speech-envelope modulated noise and speech competitors of the same gender speakers	Significant differences between groups for speech comprehension in poor masking conditions. MCI and dementia performed worse than HC and possible MCI. Older adults with MCI and dementia have difficulties in understanding speech in the presence of background distractions.
**79**	Diversity in verbal fluency performance and its associations with MRI-informed brain age matrices in normal ageing and neurocognitive disorders	Lu et al.,	2023	488 older adults, 56 with high performance, mean age 69.2 years; 205 with normal aging, mean age 70.1 years; 204 with MCI, mean age 71 years; and 23 with dementia, with a median age range of 74.2 years	Psychometric and morphometric approach to describe VF performance. Participants were asked to overtly generate as many words as possible in 60 s. First, the CVFT was performed using three trials: all participants were asked to produce words in the categories of animals, fruits, and vegetables. Second, the number of words produced in each category in 30 and 60 seconds was recorded.	Speed-based measures showed extensive and stronger associations with other cognitive functions than capacity-based measures. CVFT capacity was significantly related to younger brain age in MCI patients. CVFT capacity scores decreased in coordination with disease severity. Traditional CVFT measures at 30 and 60 seconds had modest power to differentiate high-performing older adults from normal ones and a higher degree of differentiation between MCI and high-performing individuals. Slower speeds in CVFT at 30 and 60 seconds correlated with volumetric decrease in gray matter in the left temporal pole.
**80**	Breaking the flow of thought: Increase of empty pauses in the connected speech of people with mild and moderate Alzheimer's disease	Lofgren et al.,	2022	21 patients with mild AD, mean age 70.1 years; 19 with moderate AD, mean age 70.8 years; 40 HC, mean age 67.5 years	Image description task (cookie theft) to analyze pauses within a sentence. HAM-D, BDAE	Pause frequency differentiated controls from both AD groups, while fillers did not distinguish any group. The two AD groups exhibit distinct patterns compared to HC, while none of the measures differentiated between the AD groups. Moderate AD differs from HC in initial clause pauses. Mild AD differs from controls in position within the clause. Mild AD showed a significant negative correlation between pause frequency in spontaneous speech and VF measures.

**81**	Language Impairment in Alzheimer's Disease-Robust and Explainable Evidence for AD-Related Deterioration of Spontaneous Speech Through Multilingual Machine Learning	Lindsay et al.,	2021	154 participants, 78 HC and 76 AD, from two different languages, 106 English speakers, 47 French speakers.	Image description task, responses recorded and transcribed manually. Semantic, syntactic, and paralinguistic features were extracted using NLP.	Semantic features are more generalizable. Multilingual speech shows the same error distribution in English and French. Task-specific and semantic features show similar patterns, with lower means in AD than HC. 30% of task-specific features, 28% of semantic, 39% of syntactic, and 65% of paralinguistic features are not significant, in both English and French. Using linguistic and paralinguistic features achieved a classification accuracy of 87.5%.
**82**	Language Differences Among Individuals with Normal Cognition, Amnestic and Non-Amnestic MCI, and Alzheimer's Disease	Liampas et al.,	2023	1607 participants without cognitive decline, 146 with aMCI, 92 with naMCI, and 79 with AD. Mean age 73.82 years.	Assessment included naming, comprehension, repetition, and a composite linguistic index, plus semantic and phonemic verbal fluency (60 s trials), Greek verbal learning and memory tests, BDAE and BNT short forms, complex ideational material, and a Greek vocabulary multiple-choice test.	AD and aMCI showed lower performance in composite and individual linguistic indices. The largest differences were observed between HC and AD. naMCI obtained similar results to HC. AD=aMCI in verbal repetition. Among aMCI, multidomain individuals consistently performed poorer in VF compared to single-domain individuals.
**83**	Spoken discourse in episodic autobiographical and verbal short-term memory in Chinese people with dementia: the roles of global coherence and informativeness	Kong et al.,	2023	Spoken samples from 104 subjects, subgroup of 70 personal narratives. Age range between 60 and 90 years.	Personal narratives, "Tell me about the happiest event in your life." Sequential description of images, telling a story represented in 4 cards.	Global coherence was a significant predictor of episodic autobiographical memory, explaining more than half of the variance. In the personal narrative task, global coherence positively correlated with episodic autobiographical memory. Empty speech indices, global coherence in personal narrative alone explained 61% of the variance in episodic autobiographical memory.
**84**	Sensitive Measures of Cognition in Mild Cognitive Impairment	Klooster et al.,	2021	56 HC, mean age 72.84 years; 35 aMCI, mean age 72.68 years; 28 MCI.	Precision tasks of figurative language (metaphors, verbal analogy). Sense enumeration tasks, word association tasks, multiple-choice metaphor tasks, verbal analogy tasks. CRAFTVRS, UDSBENTC; DIGFORCT; DIGBACCT; UDSVERFC; MINT	In experimental semantic and figurative language tests, aMCI showed lower performance than HC. Significant effect of sense task performance on group difference. HC outperformed aMCI in word association. Literal metaphor performance differentiated HC and aMCI. Experimental measures better differentiated the groups than standardized tests. Cortical thickness in BA35 was associated with sense task performance; a 1mm increase was associated with a 0.77% increase in senses produced by HC. This relationship was not observed in aMCI. Across all groups, mean thickness of BA35 correlated with WAT word association performance. In metaphor tasks, mean thickness of BA35 tended to predict accuracy in all groups.
**85**	Impaired Speaking-Induced Suppression in Alzheimer's Disease	Kim et al.,	2023	20 patients with AD, average age 63.77 years, and 12 controls with an average age of 63.68 years	Speaking condition: pronounce the sound "Ah" when a dot appeared on the projection screen and stop pronouncing upon receiving a visual signal to halt. Listening condition: hear a	Post hoc analyses showed significant group differences in M100 amplitude during speech production, but not during listening or in M200. AD patients exhibited reduced speech-induced suppression (SIS) in M100, mainly in the right hemisphere, due to non-suppressed M100 peaks during speech, while healthy controls showed normal SIS in both hemispheres.

					playback of analytical feedback. They had heard during the previous test, allowing the isolation of specific speech activity. MEG	
**86**	Non-transcription analysis of connected speech in mild cognitive impairment using an information unit scoring system	Kim et al.,	2022	15 subjects with aMCI, average age 72.4 years, and 15 HA, average age 72.2 years, monolingual Korean speakers	Narration of family and novel stories. Each story was presented sequentially in audio and with images. Then, participants recounted the part of the story they could remember.	Greater difference was observed in the family story compared to the novel story between the two groups. Analysis without transcription for connected speech measurement has shown its ease of use for clinicians but has not been applied to studies in mild cognitive impairment. Subtle speech impairment in individuals with mild cognitive impairment can be captured through tasks of narrating a familiar story. The information unit scoring system used within storytelling tasks is a reliable and valid measure for differentiating individuals with mild cognitive impairment from cognitively healthy adults.
**87**	Classification of Alzheimer's Disease Leveraging Multi-Task Machine Learning Analysis of Speech and Eye-Movement Data	Jang et al.,	2021	79 patients with mild to moderate AD, aMCI, or subjective memory complaint (SMC) with an average age of 72 years, and 83 controls with an average age of 65.63 years.	Pupil calibration, image description, paragraph reading, and memory recall. IReST	Novel tasks with similar accuracy to established tasks. Fusion of multimodal data across tasks resulted in a global AUC of 0.83%. Pupil calibration and memory description achieved classification accuracy AUC of 0.71% and 0.78%, respectively. Patients showed greater variation and had more eye movements, with a shorter average fixation duration than HC.
**88**	Discriminating speech traits of Alzheimer's disease assessed through a corpus of reading task for Spanish language	Ivanova et al.,	2021	Elderly native speakers of European Spanish over 60 years old, with a minimum of 6 years of primary education. HC 197, aMCI 91 (non-degenerative 73 and preclinical AD, 13), AD 74.	Standardized reading based on the first paragraph (126 syllables) of the globally renowned novel by Miguel de Cervantes, "Don Quixote of La Mancha." All participants read the text in 48-point font from a screen in a soundproof room in this language.	Individuals with preclinical and mild AD show slower, less fluent speech with more pauses, longer syllables, irregular syllabic intervals, and greater variability. Acoustic changes include lower speech energy, dysphonia, amplitude fluctuations, and reduced intensity, especially in women. Compared to healthy elderly and MCI, AD speech is monotonous, interrupted, stuttered, and imprecise, with progressive decline paralleling cognitive deterioration.
**89**	Automated analysis of propositional idea density in older adults	Hill et al.,	2021	3,316 participants, 853 men, and 2,463 women. Average age of 63.5 years.	More than half had a family history of dementia. No standardized tests are reported. Autobiographical task Participants were instructed to provide a brief autobiography.	The mean propositional idea density (PID) was higher in women. Advanced age and being male were significantly associated with decreased PID. Lower PID is associated with a higher risk of AD.

**90**	Lexical Speech Features of Spontaneous Speech in Older Persons with and Without Cognitive Impairment: Reliability Analysis	Hamrick et al.,	2023	39 adults, 29 HC, 10 with aMCI, average age 81.1 years.	Three image description tasks and two expository tasks were used to elicit speech. Speech audio was recorded and transcribed manually during a single study visit lasting approximately 75 minutes. Controlled Oral Word Association Test, Animal Naming Test and Boston Naming. Hopkins Verbal Learning Test–Revised.	Correlation strength between speech features was similar across aMCI and healthy controls, with some features (speech rate, Brunet index, filler words) showing high reliability, while others (empty words, articles, determiners, pronouns) were less reliable. Intraindividual variability differed by feature and group, being lower for speech rate and filler rate in healthy controls, and higher for articles, empty words, and pronouns depending on the group.
**91**	Development of digital voice biomarkers and associations with cognition, cerebrospinal biomarkers, and neural representation in early Alzheimer's disease	Hajjar et al.,	2023	206 participants (51% African American), of which 92 participants without cognitive impairment (40 Aβ+), average age 63.2 years, and 114 with cognitive impairment (63 Aβ+), average age 64.9 years	Connected speech was captured through picture description, natural unstructured speech, and speech during phonemic verbal fluency and confrontation naming tasks. The picture description task used the image "Circus Procession." Hopkins Verbal Learning Test-Revised, BNT (15-item), timed PF.	Lexical-semantic and acoustic scores showed higher diagnostic performance in detecting aMCI (AUC 0.80 and 0.77) compared to the BNT (AUC 0.66). Only lexical-semantic scores detected Aβ+ status. Acoustic scores were associated with hippocampal volume, while lexical-semantic scores were associated with Aβ and CSF. Both measures were significantly associated with disease progression over 2 years. Evaluation with traditional tests had false positives of up to 25%.
**92**	The MINT Sprint 2.0: A picture naming test for detection of naming impairments in Alzheimer's disease and in preclinical AD	Gollan et al.,	2024	190 English monolingual participants, 52 with dementia, average age 79 years, 61 HC, average age 77.3 years, 26 with AD, average age 80 years.	MINT sprint 2.0 naming test	The AUC values for all measures were in the good range of 0.80 or higher, with sensitivity and specificity exceeding 0.85. Participants with preclinical AD achieved fewer correct scores than HC.
**93**	Harnessing acoustic speech parameters to decipher amyloid status in individuals with mild cognitive impairment	Garcia et al.,	2023	52 patients with aMCI, average age 73 years, 65% women, 57% Aβ+.	Speech task supervised by a neuropsychologist. Description of the cookie theft image. Voice was automatically recorded. Assessments were conducted in Spanish; ambient noise was removed.	A model based on acoustic variables achieved an accuracy of 75% with an AUC of 0.79% in predicting amyloid status. 5 out of 85 acoustic characteristics showed significant differences between the groups. Models based on demographic and neuropsychological variables performed poorly with AUC < 0.70 and accuracy close to 60%. The measures that best predicted amyloid+ were those based on frequency: voice variation coefficient, average voice, average without voice, average with voice, and spectral flux; and those based on time: voice segment length x seconds.

**94**	Linguistic Biomarkers for the Detection of Mild Cognitive Impairment	Gagliardi et al.,	2021	80 subjects, 40 with conversational data, 43 with image recordings, 16 with both.	Participants completed three semi-spontaneous speech tasks via video chat—FIGURE (image description), WORK (typical workday), and DREAM (last remembered dream)—after a standard cognitive assessment.	Linguistic markers significantly improve the AUC score for predicting aMCI, from 0.80 to 0.97. By combining image markers with linguistic features, an AUC score of 0.98 is achieved.
**95**	A remote speech-based AI system to screen for early Alzheimer's disease via smartphones	Fristed et al.,	2022	133 subjects with Aβ+, 71 without dementia, 62 with aMCI or mild AD, aged 67-71 years.	Story recall administered via smartphones. ASRT includes 18 variants of short stories and 18 of long stories (average of 119 and 224 words per story, and stimulus duration ≈1 minute and 1 minute 40 seconds, respectively). Reminding Test, Category Fluency.	An AI system predicted aMCI and mild AD with an AUC of 0.85 and AB with an AUC of 0.62 in the full sample. It predicted AB in clinical subsamples of aMCI and mild AD with an AUC of 0.78. Long stories and late narratives yielded similar results.
**96**	Language performance as a prognostic factor for developing Alzheimer's clinical syndrome and mild cognitive impairment: Results from the population-based HELIAD cohort	Folia et al.,	2023	921 CN with an average age of 72.75 years and 29 MCI, average age 77.4 years	Assessment included semantic and phonemic fluency, confrontation naming, comprehension, and verbal repetition using the Greek BDAE short form and BNTsf, with participants generating category- and letter-based words, and a three-year follow-up.	In normal controls (NC), higher language composite scores reduced the risk of MCI (49%) and dementia (32%). FVS and BNTsf performance independently predicted lower risk. In MCI, only semantic fluency (FS) and naming showed significant predictive value, with each additional FS response reducing MCI risk by 16%. Other measures (comprehension, repetition, PF, SF-PF discrepancy) were not predictive. Among 955 participants, 34 developed dementia and 160 developed MCI, with converters being older, less educated, and scoring lower on MMSE and language tests.
**97**	Increasing access to cognitive screening in the elderly: Applying natural language processing methods to speech collected over the telephone	Diaz-Asper et al.,	2022	91 elderly English speakers, diagnosed with mild AD, aMCI, or HC	Speech recorded over the phone, a 20-minute interview. Participants generated as many words in the animal category as possible in 1 minute and described a favorite childhood memory.	NLP methods outperformed traditional evaluators in distinguishing AD, aMCI, and healthy controls (HC) using phone-obtained speech data. Key discriminative features included speech impoverishment measures and semantic fluency (unique animals). The ML model achieved 87% accuracy and AUC = 0.88.
**98**	A Comparison of Connected Speech Tasks for Detecting Early Alzheimer's Disease and Mild Cognitive Impairment Using Natural Language Processing and Machine Learning	Clarke et al.,	2021	50 participants, 25 HC with a mean age of 63 years, and 25 with EA or aMCI with a mean age of 71 years.	5 connected speech tasks: image description, conversational map reading task, recall of a learned narrative, procedural recall, and narration of a wordless picture book. Image description (PD); Conversational speech (CS); Narrative recall about learned material (ONR); Procedural recall (PR); and Novel narrative recount (NNR).	Performance varied with an accuracy for classifying HC vs. AD and aMCI ranging from 62% using the wordless picture book narration to 78% using narrative features about learned material. Image description achieved an accuracy of 0.76 and specificity of 0.82 in the ONR condition (narrative recall about learned material).

**99**	Correlation between Ratios of Word-finding Behavior and CIU in Patients with Mild Cognitive Impairment and Dementia of Alzheimer's Type	Choi et al.,	2022	87 participants, 31 HC with a mean age of 72.9 years, 29 aMCI with a mean age of 71.2 years, and 27 AD with a mean age of 73.6 years.	Spontaneous speech through image description tasks and word search behavior, including word reformulations, repetitions, empty words, time fillers, insertions, and delays. The images used were a cookie theft image and a continuous image of a couple arguing.	Significant differences were found in the global index (proportion of nonsensical words, repetitions, additions, and total word search behavior scores), generally in people. The relationship between word search behavior output rate and correct information units (CIU) was not significant overall. However, a significant negative relationship was found in aMCI and AD. The word search behavior outcome rate significantly predicted CIU rate in the aMCI and AD groups.
**100**	A finer-grained linguistic profile of Alzheimer's disease and Mild Cognitive Impairment	Chapin et al.,	2022	105 subjects divided into MCI, mild or moderate AD, and HC	Description of images. BDAE	The groups did not differ in generic linguistic variables such as the number or length of utterances. Compared to HC, AD produced fewer embedded clauses, indefinite nominal phrases, and aspect marking with a moderate to large effect size. Language impairment in AD and MCI at the level of specific linguistic features such as narrative specifiers and episodic richness was independent of episodic memory. AD differed significantly from HC in producing more definite pronouns. The proportion of pronouns was significantly higher in AD than in HC. The proportion of adverbial clauses was significantly lower in AD compared to HC, and the proportion of relative clauses was significantly higher in AD compared to HC.
**101**	A Linguistic Profile of Narrative Speech in Early and Late-Onset Alzheimer's Disease	Can et al.,	2021	4 groups, 62 subjects with AD, 23 with EAD (Early-Onset Linguistic Profile), average age 59.8 years, and 39 with LAD (Late-Onset), average age 78.5 years, 26 HC.	Image Story Sequence Test and Subject-Based Narration Test: Image description, narration where the examiner asked them to tell the happiest or most depressing moment of their lives. They were expected to recount their memories in a logical sequence of events. Picnic Image Description Test from the Western Aphasia Battery Revised: Cookie Theft Description Test from BDAE	A statistically significant difference was found with 95% confidence in all tests in terms of average sentence length between the groups. The EAD, LAD, and AD groups used shorter sentences compared to HC. The comparison between EAD and LAD only revealed statistically significant differences in the cookie theft description test. On average, the EAD group performed worse compared to the LAD group and formed shorter sentences. In all tests, the LAD group used more sentences than the EAD and HC groups. The EAD group spoke more in the subject-based narration test and less in the Picnic image description test. The LAD group spoke more in the Picnic image description test and less in the narration test.
**102**	Linguistic features and automatic classifiers for identifying mild cognitive impairment and dementia	Calza et al.,	2021	98 participants, 48 HC with an average age of 61.6 years, and 48 with cognitive impairment (CI), divided into 16 with amnestic mild cognitive impairment (aMCI), 16 with multidomain MCI, with an average age of 64.34 years, and 16 with early	Spontaneous speech during the execution of 3 mnemonic tasks, linguistically elicited by: "Could you describe this picture?" (figure), "Could you describe a typical workday?" (work), "Could you describe the last dream you remember?" (dream).	Good performance of classifiers when trained with manually verified data (MCC) and all tasks, slight difference in performance exhibited by the fully automatic procedure to derive all features automatically (70.5%) compared to classifier results when trained with manually verified data (74.5%). Acoustic and syntactic features confirm their good ability to distinguish subjects with MCI from controls, as already evidenced by statistical significance. 5 of the 7 rhythmic features are not far from the significance threshold. A complete linguistic profile of pathological verbal productions through computational techniques, despite their time-consuming nature, is an essential preliminary step for the implementation of a valid and reliable dementia detection tool. From a linguistic perspective, typological differences (e.g., acoustic, morphological, syntactic, and lexical levels) could strongly limit the extension of the results, making it difficult to disseminate similar tools in different geographical areas.

				dementia, with an average age of 66.38 years		
**103**	Predicting Scores on the Mini-Mental State Examination (MMSE) from Spontaneous Speech.	Bueno-Cayo et al.,	2022	33 elderly participants without a diagnosis of dementia. 20 HC and 13 aMCI. Age range between 60 and 95 years	Providing information about recent and future events through questions: "What are your plans for today and tomorrow?"	High MMSE scores showed high lexical density, discourse length, as well as the number of tokens related to time, space, and action in speech. HC had a higher score in lexical density and discourse length, and a higher number of tokens related to time, place, and action; however, only lexical density reached statistical significance. Lexical density positively correlated with MMSE scores, the latter showing a strong negative correlation with age.
**104**	Importance of Task Selection for Connected Speech Analysis in Patients with Alzheimer's Disease from an Ethnically Diverse Sample	Bose et al.,	2022	6 Bengalis with probable AD and 8 Bengali-native and sequential Bengali English bilinguals. Average age of 66.5 years.	2 connected speech tasks, Frog Story and description of picnic scene images from the Western Aphasia Battery-Revised. BMSE	The Frog Story captured specific differences between the groups and linguistic differences at 6 levels: speed, disfluencies, syntax, lexical, morphological, and semantic, while the description of images only captured 3. In the Frog Story, individuals with AD showed significant differences compared to HC in all 6 linguistic levels, exhibiting a slower speech rate with more disfluencies, characterized by more attempts at reformulation.
**105**	Comparing Pre-trained and Feature-Based Models for Prediction of Alzheimer's Disease Based on Speech	Balagopalan et al.,	2021	156 adults over 50 years old, 78 with AD and 78 HC.	Description of images, of the cookie theft.	Feature-based approaches like the BERT model significantly outperform the baseline linguistic model. BERT achieved an accuracy of 81.1% in classifying AD/non-AD. 87 features are significantly different between the two groups, with 79 being lexical-semantics and 8 being acoustic. After Bonferroni correction, only 13 are significantly different between AD and non-AD speech, and none are acoustic.
**106**	Using a Discourse Task to Explore Semantic Ability in Persons with Cognitive Impairment	Antonsson et al.,	2021	40 subjects with DC, average age 74.9 years, and 28 HC, average age 69.5 years, 27 CS (cognitively stable) 68.6 years old, Swedish as their first language.	BNT, I-Flex Discourse task modeled after the "Trip to Stockholm" task. Participants were asked to describe how they would prepare for and execute a trip to Stockholm. The instructions were as follows: participants were asked to think and plan aloud. Imagine they are going on vacation in a week. Describe all plans for 5 minutes. Boston Naming Test, verbal fluency for letters F-A-S	Significant differences were found between the groups in terms of the number of pauses, silences, fillers, false starts, and self-interruptions, but they did not surpass Bonferroni correction. The combination of SVF results and disfluency features proved to be more useful. The oral lexical retrieval test successfully differentiated between the cognitively stable and declining groups. The proportion of unrelated speech was the only measure that could differentiate between both groups.
**107**	Linguistic, visuospatial, and kinematic writing characteristics in cognitively	An et al.,	2023	31 subjects with aMCI and Aβ+, average age 72.23 years, 26 with AD, average age	Dictation of 60 regular and irregular words, the top 30% of high-frequency words and the bottom 30% of low-frequency words. Horizontal transverse	As CI progresses, performance decreases in dictation of irregular words, with errors in grapheme substitution observed. aMCI showed frequent self-corrections, involving rewriting of letters during the task. In kinematics, both aMCI and AD showed low writing speed compared to HC.

	impaired patients with beta-amyloid deposition			70.33 years, 33 HC, average age 71.24 years.	writing, longitudinal vertical writing, and mixed writing. An additional copying task was performed where participants imitated the visually presented letter as they perceived it.	
**108**	Screening for early Alzheimer's disease: enhancing diagnosis with linguistic features and biomarkers	Chou, et al.,	2024	80 participants classified as early AD (75.02 years) and normal cognition (72.23 years).	Image description task depicting Taiwanese culture.	In the early AD group, mean utterance length and mean sentence length were significantly shorter than in NC, with longer pauses (>2 seconds) and significant differences in pronoun use. A combined approach using linguistic features and biomarkers improved classification model performance. Mean utterance length was the most influential predictor, outperforming hippocampal volume in distinguishing the groups.
**109**	Language Markers of Dementia and Their Role in Early Diagnosis of Alzheimer's Disease: Exploring Grammatical and Syntactic Competence via Sentence Repetition	Kaltsa et al.,	2024	150 participants were divided into healthy young speakers, healthy older speakers without cognitive impairment, speakers with subjective cognitive decline, speakers with MCI, and speakers with mild to moderate AD, with a mean age of 61.8 years.	Verbal and Semantic Fluency Test (FAS). Litmus SRT	Grammaticality decreased significantly in the MCI and AD groups, with AD participants showing lower response accuracy. The Completeness index dropped significantly in AD, while the Accuracy index was the most sensitive measure for detecting cognitive decline.

MMSE: Mini-Mental State Examination; MOCA: Montreal Cognitive Assessment; MRI: Magnetic resonance imaging; CDR Clinical Dementia Rating; CAT Computerized axial tomography; PET Positron Emission Tomography; SPECT: Single-photon emission computed tomography; BDAE: Boston Diagnostic Aplasia Examination; BNT: Boston Naming Test; LASSI-L: Semantic Interference and Learning; EGG: Electroencephalogram; HC: Healthy control; LCR: Liquido cefaloraquideo; MINT: The Multilingual Naming Test; IReST: International Reading Speed Test; MEG: Magnetoencefalogram; CRAFTVRS: Craft Story Verbatim; UDSVERFC:Verbal Fluency Phonemic Test 'F"; UDSVERLC: Verbal Fluency Phonemic Test 'L'; CVFT: Category Verbal Fluency Test; FAS COWA: FAS Word Fluency o Controlled Oral Word Associacion-COWA; IMT: The Implicit Management Test: QCS: Quebec Semantic Questionnaire; WAY2AGE: Azure voice-bot for screening cognitive impairment; R-AVLT: The Rey Auditory Verbal Learning Test; CCLNT: the Copenhagen Cross-Linguistic Naming Test; CD:Cognitive decline; NC: normal cognition.
