# Supplementary material for: Assessment of Language Impairments Towards Identifying Markers for Early Diagnosis of Pathological Cognitive Decline
Source: Behav Sci (Basel). 2026 Feb 28;16(3):345. doi: 10.3390/bs16030345 (PMC13024424; doi:10.3390/bs16030345)
Supplement: Supplementary file 1 [file behavsci-16-00345-s001.zip › Table S3.pdf]

Table S3: Quality Scoring of Quantitative Studies

CRITERIA		YES<br>(2)	PARTIAL<br>(1)	NO<br>(0)
1	**Question or objective suffi ciently described?**	Is easily identifi ed in the introductory section (or fi rst paragraph of methods section). Specifi es (where applicable, depending on study design) all of the following: purpose, subjects/target population, and the specifi c intervention(s)/association(s)/descriptive parameter(s) under investigation. A study purpose that only becomes apparent after studying other parts of the paper is not considered suffi ciently described.	Vaguely/incompletely reported (e.g. “describe the effect of” or “examine the role of” or “assess opinion on many issues” or “explore the general attitudes”...); or some information has to be gathered from parts of the paper other than the introduction/background/objective section.	Question or objective is not reported, or is incomprehensible.
2	**Design evident and appropriate to answer study question?**	Design is easily identifi ed and is appropriate to address the study question /objective.	Design and /or study question not clearly identifi ed, but gross inappropriateness is not evident; or design is easily identifi ed but only partially addresses the study question.	Design used does not answer study question (e.g., a comparison group is required to answer the study question, but none was used); or design cannot be identifi ed.
3	**Method of subject selection (and comparison group selection, if applicable) or source of information/input variables (e.g., for decision analysis) is described and appropriate.**	Described and appropriate. Selection strategy designed (i.e., consider sampling frame and strategy) to obtain an unbiased sample of the relevant target population or the entire target population of interest (e.g., consecutive patients for clinical trials, population-based random sample for case-control studies or surveys). Where applicable, inclusion/exclusion criteria are described and defined. Studies of volunteers: methods and setting of recruitment reported. Surveys: sampling frame/strategy clearly described and appropriate.	Selection methods (and inclusion/exclusion criteria, where applicable) are not completely described, but no obvious inappropriateness. Or selection strategy is not ideal (i.e., likely introduced bias) but did not likely seriously distort the results (e.g., telephone survey sampled from listed phone numbers only; hospital based case-control study identifi ed all cases admitted during the study period, but recruited controls admitted during the day/evening only). Any study describing participants only as “volunteers” or “healthy volunteers”. Surveys: target population mentioned but sampling strategy unclear.	No information provided. Or obviously inappropriate selection procedures. Or presence of selection bias which likely seriously distorted the results (e.g., obvious selection on “exposure” in a case-control study).
4	**Subject (and comparison group, if applicable) characteristics or input variables/information (e.g., for decision analyses) suffi ciently described?**	Suffi cient relevant baseline/demographic information clearly characterizing the participants is provided. Where applicable, reproducible criteria used to describe/categorize the participants are clearly defi ned. If “healthy volunteers” are used, age and sex must be reported (at minimum). Decision analyses: baseline estimates for input variables are clearly specifi ed.	Poorly defi ned criteria (e.g. “hypertension”, “healthy volunteers”, “smoking”). Or incomplete relevant baseline / demographic information (e.g.,information on likely confounders not reported). Decision analyses: incomplete reporting of baseline estimates for input variables.	No baseline / demographic information provided. Decision analyses: baseline estimates of input variables not given.
5	**If random allocation to treatment group was possible, is it described?**	True randomization done - requires a description of the method used (e.g., use of random numbers).	Randomization mentioned, but method is not (i.e. it may have been possible that randomization was not true).	Random allocation not mentioned although it would have been feasible and appropriate (and was possibly done).

**6**	**If interventional and blinding of investigators to intervention was possible, is it reported?**	Blinding reported.	Blinding reported but it is not clear who was blinded.	Blinding would have been possible (and was possibly done) but is not reported.
**7**	**If interventional and blinding of subjects to intervention was possible, is it reported?**	Blinding reported.	Blinding reported but it is not clear who was blinded.	Blinding would have been possible (and was possibly done) but is not reported.
**8**	**Outcome and (if applicable) exposure measure(s) well defi ned and robust to measurement / misclassifi cation bias? Means of assessment reported?**	Defi ned (or reference to complete defi nitions is provided) and measured according to reproducible, "objective" criteria. Little or minimal potential for measurement /misclassifi cation errors. Surveys: clear description (or reference to clear description) of questionnaire/interview content and response options.<br>Decision analyses: sources of uncertainty are defi ned for all input variables.	Definition of measures leaves room for subjectivity, or not sure (i.e., not reported in detail, but probably acceptable). Or precise defi nition(s) are missing, but no evidence or problems in the paper that would lead one to assume major problems. Or instrument/mode of assessment(s) not reported. Or misclassifi cation errors may have occurred, but they did not likely seriously distort the results (e.g., slight diffi culty with recall of long-ago events; exposure is measured only at baseline in a long cohort study). Surveys: description of questionnaire/interview content incomplete; response options unclear. Decision analyses: sources of uncertainty are defi ned only for some input variables.	Measures not defi ned, or are inconsistent throughout the paper. Or measures employ only ill-defi ned, subjective assessments, e.g. "anxiety" or "pain." Or obvious misclassifi cation errors/measurement bias likely seriously distorted the results (e.g., a prospective cohort relies on self-reported outcomes among the "unexposed" but requires clinical assessment of the "exposed"). Surveys: no description of questionnaire/interview content or response options. Decision analyses: sources of uncertainty are not defi ned for input variables.
**9**	**Sample size appropriate?**	Seems reasonable with respect to the outcome under study and the study design. When statistically signifi cant results are achieved for major outcomes, appropriate sample size can usually be assumed, unless large standard errors (SE > ½ effect size) and/or problems with multiple testing are evident. Decision analyses: size of modeled cohort / number of iterations specifi ed and justifi ed.	Insuffi cient data to assess sample size (e.g., sample seems "small" and there is no mention of power/sample size/effect size of interest and/or variance estimates aren't provided). Or some statistically signifi cant results with standard errors > ½ effect size (i.e., imprecise results). Or some statistically signifi cant results in the absence of variance estimates. Decision analyses: incomplete description or justifi cation of size of modeled cohort / number of iterations.	Obviously inadequate (e.g., statistically non-signifi cant results and standard errors > ½ effect size; or standard deviations > _ of effect size; or statistically non-signifi cant results with no variance estimates and obviously inadequate sample size). Decision analyses: size of modeled cohort / number of iterations not specifi ed.
**10**	**Analysis described and appropriate?**	Analytic methods are described (e.g. "chi square"/ "t-tests"/"Kaplan-Meier with log rank tests", etc.) and appropriate.	Analytic methods are not reported and have to be guessed at, but are probably appropriate. Or minor fl aws or some tests appropriate, some not (e.g., parametric tests used, but unsure whether appropriate; control group exists but is not used for statistical analysis). Or multiple testing problems not addressed.	Analysis methods not described and cannot be determined. Or obviously inappropriate analysis methods (e.g., chi-square tests for continuous data, SE given where normality is highly unlikely, etc.). Or a study with a descriptive goal / objective is over-analyzed.

11	**Some estimate of variance (e.g., confi dence intervals, standard errors) is reported for the main results/outcomes (i.e., those directly addressing the study question/objective upon which the conclusions are based)?**	Appropriate variances estimate(s) is/are provided (e.g., range, distribution, confi dence intervals, etc.). Decision analyses: sensitivity analysis includes all variables in the model.	Undefi ned "+/-" expressions. Or no specifi c data given, but insuffi cient power acknowledged as a problem. Or variance estimates not provided for all main results/outcomes. Or inappropriate variance estimates (e.g., a study examining change over time provides a variance around the parameter of interest at "time 1" or "time 2", but does not provide an estimate of the variance around the difference). Decision analyses: sensitivity analysis is limited, including only some variables in the model	No information regarding uncertainty of the estimates. Decision analyses: No sensitivity analysis.
12	**Controlled for confounding?**	Randomized study, with comparability of baseline characteristics reported (or non-comparability controlled for in the analysis). Or appropriate control at the design or analysis stage (e.g., matching, subgroup analysis, multivariate models, etc). Decision analyses: dependencies between variables fully accounted for (e.g., joint variables are considered).	Incomplete control of confounding. Or control of confounding reportedly done but not completely described. Or randomized study without report of comparability of baseline characteristics. Or confounding not considered, but not likely to have seriously distorted the results. Decision analyses: incomplete consideration of dependencies between variables.	Confounding not considered, and may have seriously distorted the results. Decision analyses: dependencies between variables not considered.
13	**Results reported in suffi cient detail?**	Results include major outcomes and all mentioned secondary outcomes.	Quantitative results reported only for some outcomes. Or diffi cult to assess as study question/objective not fully described (and is not made clear in the methods section), but results seem appropriate.	Quantitative results are reported for a subsample only, or "n" changes continually across the denominator (e.g., reported proportions do not account for the entire study sample, but are reported only for those with complete data -- i.e., the category of "unknown" is not used where needed). Or results for some major or mentioned secondary outcomes are only qualitatively reported when quantitative reporting would have been possible (e.g., results include vague comments such as "more likely" without quantitative report of actual numbers).
14	**Do the results support the conclusions?**	All the conclusions are supported by the data (even if analysis was inappropriate). Conclusions are based on all results relevant to the study question, negative as well as positive ones (e.g., they aren't based on the sole significant fi nding while ignoring the negative results). Part of the conclusions may expand beyond the results, if made in	Some of the major conclusions are supported by the data, some are not. Or speculative interpretations are not indicated as such. Or low (or unreported) response rates call into question the validity of generalizing the results to the	None or a very small minority of the major conclusions are supported by the data. Or negative fi ndings clearly due to low power are reported as defi nitive evidence against the alternate

		addition to rather than instead of those strictly supported by data, and if including indicators of their interpretative nature (e.g., “suggesting,” “possibly”).	target population of interest (i.e., the population defi ned by the sampling frame/strategy).	hypothesis. Or conclusions are missing. Or<br>extremely low response rates invalidate generalizing the results to the target population of interest (i.e., the population defi ned by the sampling frame/ strategy).

*Calculation of the total score: Total sum = (number of "yes" * 2) + (number of "partials" * 1). Total, possible sum = 28. Final score: total sum / total possible sum*
